# Sodium channel endocytosis drives axon initial segment plasticity

**DOI:** 10.1126/sciadv.adf3885

**Published:** 2023-09-15

**Authors:** Amélie Fréal, Nora Jamann, Jolijn Ten Bos, Jacqueline Jansen, Naomi Petersen, Thijmen Ligthart, Casper C. Hoogenraad, Maarten H. P. Kole

**Affiliations:** ^1^ Axonal Signaling Group, Netherlands Institute for Neurosciences (NIN), Royal Netherlands Academy for Arts and Sciences (KNAW), Amsterdam, Netherlands.; ^2^ Cell Biology, Neurobiology and Biophysics, Department of Biology, Faculty of Science, Utrecht University, Utrecht, Netherlands.; ^3^ Department of Neuroscience, Genentech Inc, South San Francisco, CA, USA.

## Abstract

Activity-dependent plasticity of the axon initial segment (AIS) endows neurons with the ability to adapt action potential output to changes in network activity. Action potential initiation at the AIS highly depends on the clustering of voltage-gated sodium channels, but the molecular mechanisms regulating their plasticity remain largely unknown. Here, we developed genetic tools to label endogenous sodium channels and their scaffolding protein, to reveal their nanoscale organization and longitudinally image AIS plasticity in hippocampal neurons in slices and primary cultures. We find that *N*-methyl-d-aspartate receptor activation causes both long-term synaptic depression and rapid internalization of AIS sodium channels within minutes. The clathrin-mediated endocytosis of sodium channels at the distal AIS increases the threshold for action potential generation. These data reveal a fundamental mechanism for rapid activity-dependent AIS reorganization and suggests that plasticity of intrinsic excitability shares conserved features with synaptic plasticity.

## Teaser

Rapid activity-dependent sodium channel internalization at the axon initial segment tunes the action potential output.

## INTRODUCTION

The axon initial segment (AIS) is a critical domain of all excitable neurons, which maintains neuronal polarity and functionally acts as the final synaptic integration site for action potential (AP) generation (*1*, *2*). The threshold and upstroke dynamics of APs rely, in part, on the specific distribution, density, and isoforms of voltage-gated sodium (Na_V_) channels expressed along the AIS. The AIS localization of Na_V_1.2 and Na_V_1.6 channels, the two main isoforms of Na_V_ channels in excitatory neurons, is mediated by the interaction with the AIS scaffold protein ankyrin G (AnkG) (*3*, *4*). Over the last decade, emerging evidence showed that AIS morphology changes with activity in a brain region- and cell type–dependent manner. In vivo experiments demonstrated that changes in sensory input streams induce bidirectional changes of AIS length with temporal scales of 1 hour to several weeks, mediating a homeostatic plasticity of AP output (*5*–*7*). AIS plasticity varies from subtle changes in ion channel isoform expression (*8*), to relocation of the AIS along the axis of the axonal membrane (*9*), changes in AIS length (*6*), or to a complete proteolysis of AIS proteins during excitotoxicity (*10*–*12*). However, in contrast to the detailed understanding of the mechanisms regulating synaptic plasticity (*13*), the specific molecular mechanisms controlling AIS plasticity are far from being understood. A comprehensive molecular model for how AIS plasticity is induced and expressed is still lacking. Molecular players implicated in activity-dependent AIS relocation include L-type voltage-gated calcium (Ca^2+^) channels, calcineurin, myosin II/phospho-myosin light chain, and/or the AKT-ULK1 pathway (*9*, *14*–*19*). A major limitation in our understanding of AIS plasticity mechanisms is that they mostly have been studied by comparing the AIS across large populations of differentially treated neurons. Live reporters allowing longitudinal analysis of AIS structure without affecting its organization and function are still limited (*20*, *21*). Moreover, while most studies have focused on the scaffold protein AnkG, little is known about the redistribution of Na_V_ channels during plasticity. During AIS development, two main trafficking routes have been reported for membrane proteins. They are either inserted into the somatic and/or axonal membrane before diffusing laterally until they get immobilized at the AIS, like neurofascin-186 and the voltage-gated potassium channel K_V_7.3 (*22*), or directly targeted into the AIS membrane, as has been shown for Na_V_1.6 channels (*23*). Nevertheless, in both cases, their stable accumulation at the AIS depends on the local inhibition of their internalization by the interaction with AnkG and adaptor proteins (*24*–*26*). Here, we hypothesized that, during activity-dependent AIS plasticity, the membrane channel reorganization is mediated by the dynamic trafficking of Na_V_1 channels. To resolve the molecular events underlying AIS plasticity, we took advantage of a newly developed knock-in mouse line expressing green fluorescent protein (GFP)–labeled AnkG (*27*) and used the ORANGE CRISPR-Cas9 system (*28*) to tag endogenous Na_V_1 channels, allowing the interrogation and live imaging of the molecular signaling pathways during activity-induced structural plasticity of the AIS.

## RESULTS

### NMDAR activation induces both synaptic depression and AIS plasticity

**Fig. 1.**
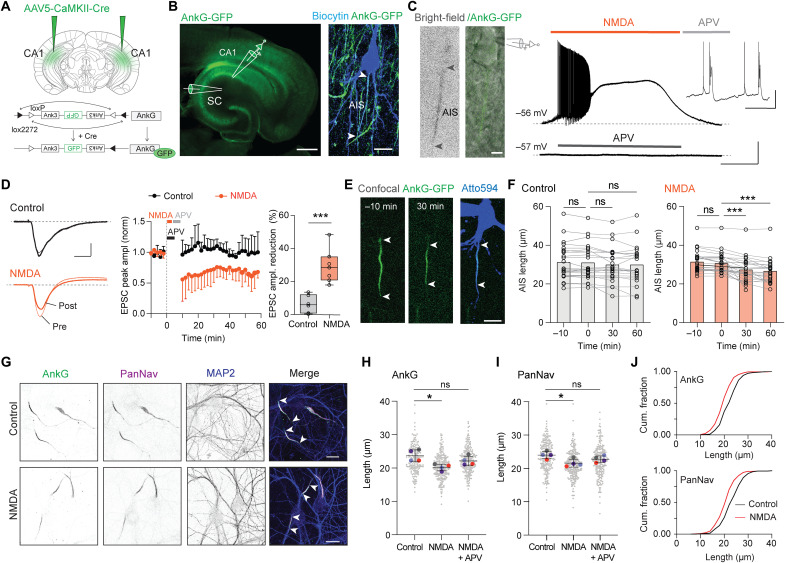
NMDAR activation induced AIS shortening in acute hippocampal slices and cultured neurons. (**A**) Genetically induced AnkG-GFP expression in CA1. (**B**) Hippocampal AnkG-GFP with patch-clamp configuration stimulating SC and CA1 pyramidal neuron filled with biocytin and immunostained against GFP. (**C**) Live bright-field and AnkG-GFP image during whole-cell recording. Top: Application of 20 μM NMDA followed by APV (“NMDA”)–induced AP burst firing. Bottom: APV alone (“control”) did not change resting potential. (**D**) Example EPSC traces before (pre) and after 60 min (post) control or NMDA treatment. NMDA causes LTD (NMDA, *n* = 7; control, *n* = 6; unpaired *t* test, ***P < 0.001). (**E**) Neuron filled with Atto594 and positive for AnkG-GFP before and after NMDA. White arrows indicate AIS start and end. (**F**) NMDA significantly reduces AIS length (mixed-effect analysis: control, *P* = 0.0254; NMDA, *P* < 0.0001. Šídák’s multiple comparisons test: control, *P* > 0.05 for 0 versus −10, 30, and 60; NMDA, −10 versus 0; *P* = 0.06 (ns), 0 versus 30; ***P < 0.001, 0 versus 60; ***P < 0.001. control: *n* = 24, 16 whole cell, 8 imaging; NMDA: *n* = 25, 14 whole cell, 11 imaging). (**G**) Immunostaining of AnkG, PanNav, and MAP2 in hippocampal neurons in control and after NMDA (4 min, 50 μM NMDA). (**H** to **I**) AnkG (H) and PanNav (I) lengths in indicated conditions (*N* = 4 cultures, *n* > 440 neurons per condition). Matched Friedman test with Dunn’s multiple comparisons test. AnkG: NMDA, *P = 0.027; NMDA + APV, *P* = 0.96 (ns). PanNav: NMDA, *P = 0.027; NMDA + APV, *P* = 0.15 (ns). (**J**) Cumulative fraction plots for AnkG and PanNav lengths. Scale bars, 500 and 15 μm (B); 10 μm; 1 min, 30 mV (C); 50 ms, 30 mV (inset of C); 100 pA, 10 ms (D); 10 μm (E); and 20 μm (G).

To image the AIS of individual pyramidal neurons in real time, we took advantage of a recently generated AnkG-GFP knock-in mouse line and performed bilateral injections with adeno-associated virus 5 (AAV5)–calmodulin-dependent protein kinase II (CamKII)–Cre into the hippocampal CA1 area to obtain Cre-dependent expression of AnkG-GFP from the CaMKII promoter (Fig. 1A) (*27*). Confocal imaging in acute hippocampal slices showed that AnkG-GFP fluorescence was detectable in the proximal axon of pyramidal neurons along the pyramidal layer (Fig. 1B), and the GFP signal colocalized with the AIS markers AnkG, β4-spectrin (fig. S1A), and Na_V_1.6 (*n* = 10 AIS; fig. S1B). To induce activity-dependent plasticity, we modified a previously developed approach for chemical long-term synaptic depression and exposed acute slices to *N*-methyl-d-aspartate (NMDA) (*29*). When 20 μM of the receptor agonist NMDA was applied for 3 min, rapidly followed by the antagonist 2-amino-5-phosphonovaltuderic acid (APV; 100 μM, 5 min), we observed a strong membrane depolarization causing transient AP firing (on average, 4.11 ± 1.35 min, *n* = 14) but not with APV alone (control, Fig. 1C), enabling correlating AnkG-GFP imaging with changes of neuronal network activity. Stimulation of the Schaffer collaterals (SCs) and recording the evoked excitatory postsynaptic currents (EPSCs) from CA1 pyramidal neurons confirmed that NMDA exposure caused long-term depression (LTD; at 60 min, on average, ~30% peak EPSC amplitude reduction in NMDA versus ~6% in control; Fig. 1, B and D). When we simultaneously imaged AnkG-GFP fluorescence during NMDA application, we observed a significant decrease in AIS length already at 30 min (*P* < 0.0001; and at 60 min, a 15% or ~4.75 μm length reduction, *P* < 0.0001; Fig. 1, E and F). Such rapid AnkG shortening is consistent with recent longitudinal imaging of AnkG-GFP in this mouse line (*27*). Also, the distance of the AIS start relative to the soma was increased in the NMDA group (*P* < 0.01, ~3 μm; fig. S1C), suggesting that both the proximal and distal regions of the AIS are putative sites of plasticity (fig. S1C). In control imaging sessions (APV alone), no AIS shortening was observed, excluding technical confounding factors by AnkG-GFP imaging or whole-cell recordings (*P* = 0.13; 0 versus 60 min; Fig. 1F). Furthermore, in separate experiments, when we examined the effects of NMDA application on AIS length in acute slices not used for electrophysiological recordings or live imaging, β4-spectrin staining confirmed that NMDA induced a 9% shortening of the AIS within 30 min (*n* > 200 AISs per condition; fig. S1D). Finally, in control experiments applying APV before, during, and after NMDA application, neither LTD nor AIS plasticity was triggered (fig. S1, E and F).

Next, we examined NMDAR-mediated AIS plasticity in cultured hippocampal neurons. We treated DIV14 (14 days in vitro) neurons with 50 μM NMDA (4 min) and determined the AIS length after 30 min recovery compared to control cultures (no NMDA). We observed a significant 20% reduction of AnkG and ~10% in PanNav (antibody recognizing all Na_V_1 isoforms), which was blocked by APV (100 μM, 3 min before and during NMDA; Fig. 1, G to J). These results are consistent with the findings obtained in acute slices. A leftward shift in the cumulative distribution plots indicated that the NMDA-induced shortening triggered a global reduction in length of the AIS in all neurons rather than a strong reduction in a subpopulation (Fig. 1J). Together, these data show that brief NMDA application, which causes a phasic increase in AP firing and triggers synaptic LTD, is also associated with the robust and rapid shortening of the AIS, both in hippocampal acute slices and cultured neurons.

### Loss of Nav following NMDAR activation specifically occurs in the distal AIS

**Fig. 2.**
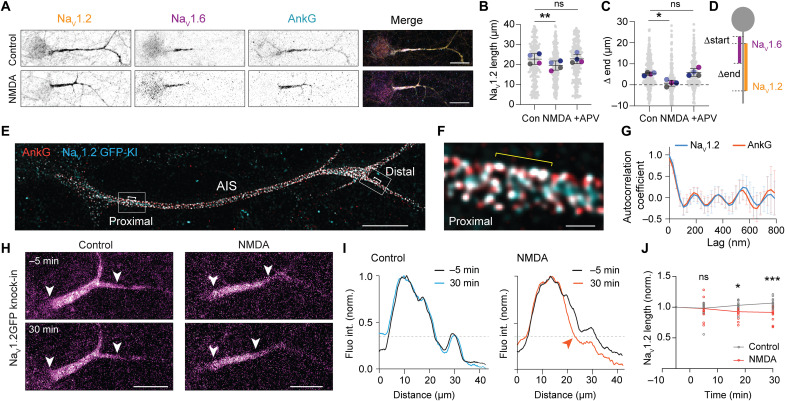
Na_V_1.2 is selectively reduced in the distal AIS during NMDAR-mediated plasticity. (**A**) Immunostaining for Na_V_1.2, Na_V_1.6 and AnkG on DIV14 in control and NMDA condition. Scale bars, 20 μm. (**B**) Average length of Na_V_1.2 was reduced by NMDA exposure and blocked by APV (NMDA + APV). Repeated-measures one-way ANOVA with Dunnett’s multiple comparisons test: NMDA, **P = 0.008; NMDA + APV, *P* = 0.99 (ns). *N* = 4 cultures with at least 236 neurons per condition. (**C** and **D**) NMDA-mediated reduction in Δ end (Na_V_1.2 to Na_V_1.6). (C) Repeated-measure one-way ANOVA with Dunnett’s multiple comparisons test, *N* = 4 cultures. NMDA, *P = 0.022; NMDA + APV, *P* = 0.76 (ns). At least 260 neurons per condition, and corresponding scheme (D). (**E** and **F**) STED image of DIV14 Na_V_1.2-GFP knock-in neuron stained for GFP and AnkG with zoom in of the proximal AIS. Scale bars, 5 μm and 500 nm. (**G**) Autocorrelation coefficient of AnkG and Na_V_1.2 fluorescence profiles in the proximal AIS, *n* = 8 neurons. (**H**) Live-cell imaging of Na_V_1.2-GFP knock-in neuron 5 min before and 30 min after NMDA and control. Scale bars, 10 μm. White arrowheads point to start and end of Na_V_1.2-GFP signal. (**I**) Corresponding fluorescence intensities normalized and smoothened over 1 μm. Dashed lines delineate the thresholds of end position, and red arrow points at the distal shortening 30 min after NMDA. (**J**) Average Na_V_1.2-GFP length of control neurons (gray, *n* = 13 neurons in *N* = 2 cultures) or in NMDA condition (red, *n* = 15 in *N* = 2 cultures) normalized to the first frame. Mixed-effects analysis with Šídák’s multiple comparisons test: 5 min, *P* > 0.99 (ns); 17.5 min, *P = 0.019; 30 min, ***P = 0.0009.

The AIS of hippocampal excitatory neurons contains both sodium channel isoforms Na_V_1.2 and Na_V_1.6 (*30*). To test whether NMDA-induced AIS plasticity selectively affects one or both isoforms, we first characterized their distribution along the AIS in primary cultured neurons. Staining of DIV14 hippocampal neurons revealed that 90 and 80% of AnkG-positive AISs were also positive for Na_V_1.2 and Na_V_1.6, respectively, with Na_V_1.2 expressed along the entire AIS, whereas Na_V_1.6 was predominantly found in the proximal AIS (fig. S2A). At DIV21, Na_V_1.6 expression became more prominent in comparison to Na_V_1.2 (fig. S2, B to D). Notably, in DIV14 cultures, brief application of NMDA significantly reduced the length of Na_V_1.2 but not Na_V_1.6 (Fig. 2, A and B, and fig. S3B), by 14% compared to control condition in ~75% of the experiments (*n* = 21 cultures), which was blocked by APV (Fig. 2B). Next, we compared the intensity and the integrated intensity of Na_V_1 isoform staining after NMDA application, normalized to the control condition in each experiment. While neither Na_V_1.6 staining mean intensity nor density changed after NMDA exposure, Na_V_1.2 staining density was reduced by 25%, with no change in the mean intensity (fig. S3, A to C). These findings suggest that the NMDAR-induced shortening may reflect a local channel removal rather than a global loss of Na_V_1.2 channels. We did not detect a shift of the AIS along the axon; the absolute start position of both Na_V_1.2 and Na_V_1.6 remained unaltered (fig. S3D). To further explore the location of NMDAR-induced changes, we next measured Na_V_1.2 and Na_V_1.6 relative start and end positions to determine the location of AIS shorting (Fig. 2, C and D, and fig. S3E). NMDAR activation changed the relative start positions of Na_V_1.6 and Na_V_1.2 (~2.2 μm reduction) and strongly affected the relative end positions (~5 μm reduction), suggesting that the length reduction of the AIS occurs at both extremities, but mostly reflects changes at the distal end of the AIS (Fig. 2C).

To directly monitor Na_V_1.2 channel membrane dynamics in living cells without overexpression artifacts, we used the ORANGE CRISPR-Cas9 system to fluorescently tag the endogenous Na_V_1.2 channel (*28*). Insertion of GFP in the C-terminal part of the channel revealed a strong enrichment of Na_V_1.2 at the AIS, while lower GFP levels could be detected in the distal axon and somato-dendritic compartment (fig. S4A), as previously reported (*31*). Using stimulated emission depletion (STED) microscopy, we observed a colocalization of AnkG with Na_V_1.2 (GFP) adopting a ring-like organization throughout the AIS (Fig. 2, E and F, and fig. S4C). The autocorrelation analysis revealed a highly periodic arrangement of the two proteins, both in the proximal and distal AIS (Fig. 2G and fig. S4, D to F) with a similar peak-to-peak distance of ~190 nm (fig. S4F), as previously observed for Na_V_1.2, Na_V_1.6, and AnkG (*31*, *32*). Moreover, NMDA application caused a ~10% reduction in the length of Na_V_1.2-GFP signal (fig. S4, A and B), indicating that our tagging approach does not impair Na_V_1.2 channel behavior during plasticity.

We then performed time-lapse imaging of endogenous Na_V_1.2-GFP before, during, and after NMDA application (Fig. 2, H to J) and observed a loss of GFP fluorescence after 30 min, specifically in the distal AIS (Fig. 2, H and I). Notably, the average normalized GFP length was already significantly reduced at 17.5 min after incubation with NMDA (~10% reduction; Fig. 2J). The comparison of Na_V_1.2 start and end positions before and after further confirmed a selective and significant reduction of Na_V_1.2 expression at the distal AIS (fig. S4G). Together, our data show converging lines of evidence for a rapid NMDAR-dependent remodeling of the AIS, mostly affecting distally localized Na_V_1.2 channels. The greater degree of plasticity in this subcellular domain implies the presence of specific mechanisms initiated by NMDAR activation and locally controlling Na_V_ isoform distribution.

### Synaptic NMDAR-mediated depolarization triggers AIS plasticity

**Fig. 3.**
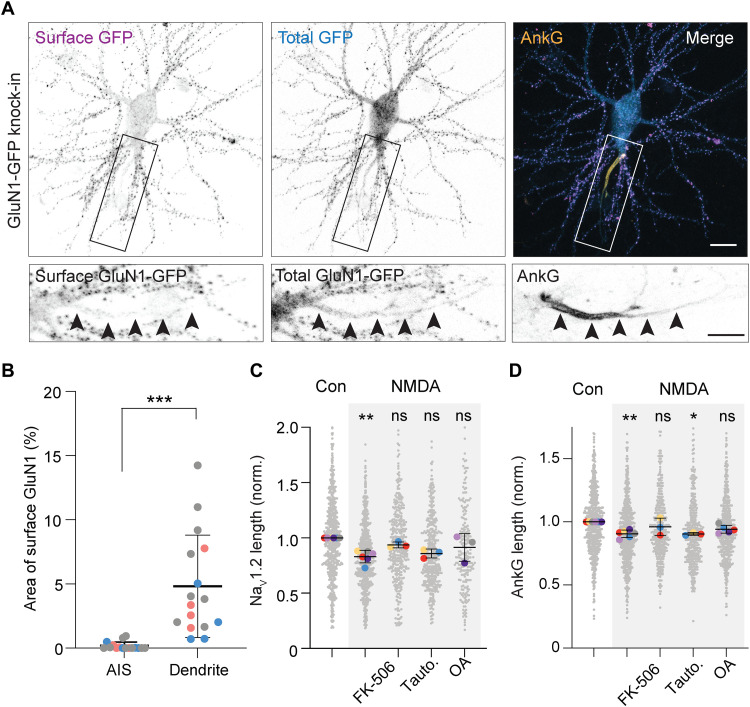
AIS plasticity is triggered by the activation of synaptic NMDARs and relies on calcineurin. (**A**) GluN1-GFP knock-in neurons stained for extracellular GFP, total GFP, and AnkG. Scale bars, 15 and 10 μm. Arrows show the trajectory of the AIS and axon. (**B**) Percentage of the AIS or dendrites surface positive for extracellular GFP. Wilcoxon’s test, ***P = 0.001, *n* = 16 neurons from three independent cultures. (**C** and **D**) Na_V_1.2 and AnkG relative lengths in NMDA and in the presence of FK-506, tautomycetin (Tauto), or okadaic acid (OA). *N* = 3 to 6 cultures, Kruskal-Wallis test. For Na_V_1.2: NMDA, **P = 0.0024; FK-506, *P* = 0.93 (ns); Tauto, *P* = 0.06 (ns); OA, *P* = 0.82 (ns). For AnkG: NMDA, **P = 0.004; FK-506, *P* = 0.99 (ns); Tauto, *P = 0.015; OA, *P* = 0.28 (ns).

Although some studies reported the presence of NMDARs at axonal presynaptic release terminals (*33*), they are typically not localized at the AIS (*34*). To unravel the precise localization of NMDARs in DIV14 primary hippocampal neurons, we used ORANGE to extracellularly tag the endogenous obligatory NMDAR subunit GluN1 with GFP (Fig. 3, A and B) (*28*). We first stained for GFP without permeabilization and only detected a very faint surface expression of GluN1 in the AIS of 50% of all neurons. When present at the AIS, membrane GFP signal only occupied 0.16% of AIS surface (~30× lower than at dendritic membrane sites; 4.8% occupancy; Fig. 3, A and B). A subsequent total GFP staining revealed a diffuse pattern in the neuron, including in the AIS, as well as bright and dense puncta in the postsynaptic density (Fig. 3A and fig. S5A). The low abundancy of NMDARs at the AIS suggests that the effect of NMDA application might be mediated via extrasynaptic or synaptic NMDARs at somato-dendritic sites. To test this idea experimentally and distinguish between these distinct NMDAR sites, we used MK-801, blocking glutamate-bound NMDARs (*35*, *36*). MK-801 applied 5 min before and during NMDA application blocked the shortening of both AnkG and Na_V_1.2, indicating that activation of synaptic NMDARs is required to trigger AIS plasticity (fig. S5, B and C). Together, the absence of NMDARs inserted at the AIS membrane and the requirement of activated NMDARs for the induction of AIS shortening reveals that the activation of synaptic NMDAR is the trigger for rapid AIS plasticity.

Which signaling pathway links synaptic NMDAR activation with AIS plasticity? In acute slice experiments we observed that NMDA caused a strong membrane depolarization with AP generation as well as LTD (Fig. 1, C and D). To test whether induction of synaptic LTD alone is sufficient to drive AIS plasticity, we performed experiments in which we evoked LTD electrically (e-LTD; SC stimulation, 900 pulses at 1 Hz; fig. S5, D and E). Although we observed strong LTD (~48% EPSC peak amplitude reduction versus 8% in control), we did not observe AIS length changes (fig. S5, F and G). To further examine the role of LTD in AIS plasticity, we used a NMDAR-independent induction protocol for LTD based on mGluR activation with the agonist (*S*)-3,5-Dihydroxyphenylglycine (DHPG) (*37*). The results showed that neither the length of AnkG, Na_V_1.2, nor Na_V_1.6 changed (fig. S5H). We next hypothesized that the postsynaptic depolarization and associated APs during NMDA application are required to trigger AIS plasticity. To test this conjecture directly, we reproduced the NMDA-induced depolarization intrinsically by injecting positive DC current, keeping the neuron for ~5 min above threshold for AP firing and live-imaged AnkG-GFP. The results showed that the AIS length remained constant (fig. S5I). Together, the experimental results show that neither synaptic depression nor somatic AP generation causes AIS plasticity, leaving open the possibility that the combination of dendritic NMDAR activation and axosomatic firing is necessary to trigger NMDAR-mediated Na_V_1.2 removal from the distal AIS.

Next, we sought to identify the molecular signaling cascade responsible for the rapid NMDAR-mediated AIS remodeling. Both LTD and NMDAR-induced intrinsic plasticity of CA1 pyramidal neuron firing cause activation of numerous phosphatases (PPs) (*29*, *38*, *39*) and some of these have also been shown to be implicated in AIS plasticity, like calcineurin (*14*). To test the involvement of calcineurin and distinct PPs, we applied NMDA in the presence of specific pharmacological inhibitors and assessed their ability to prevent the shortening of Na_V_1.2 and AnkG (Fig. 3, C and D, respectively). Inhibiting calcineurin (FK-506, 50 mM) or PP2A [with low dose of okadaic acid (OA), 2 nM] blocked the shortening of both AnkG and Na_V_1.2 induced by NMDAR activation. Inhibition of PP1 (tautomycetin, 10 μM) prevented the shortening of Na_V_1.2 but not of AnkG (Fig. 3, C and D). Thus, these results show that dephosphorylation is an important process in the removal of Na_V_1.2 at the AIS and reveal a common signaling pathway leading to the expression of LTD at synapses and the AIS shortening after activation of NMDAR.

### AIS shortening is mediated by clathrin-mediated endocytosis

**Fig. 4.**
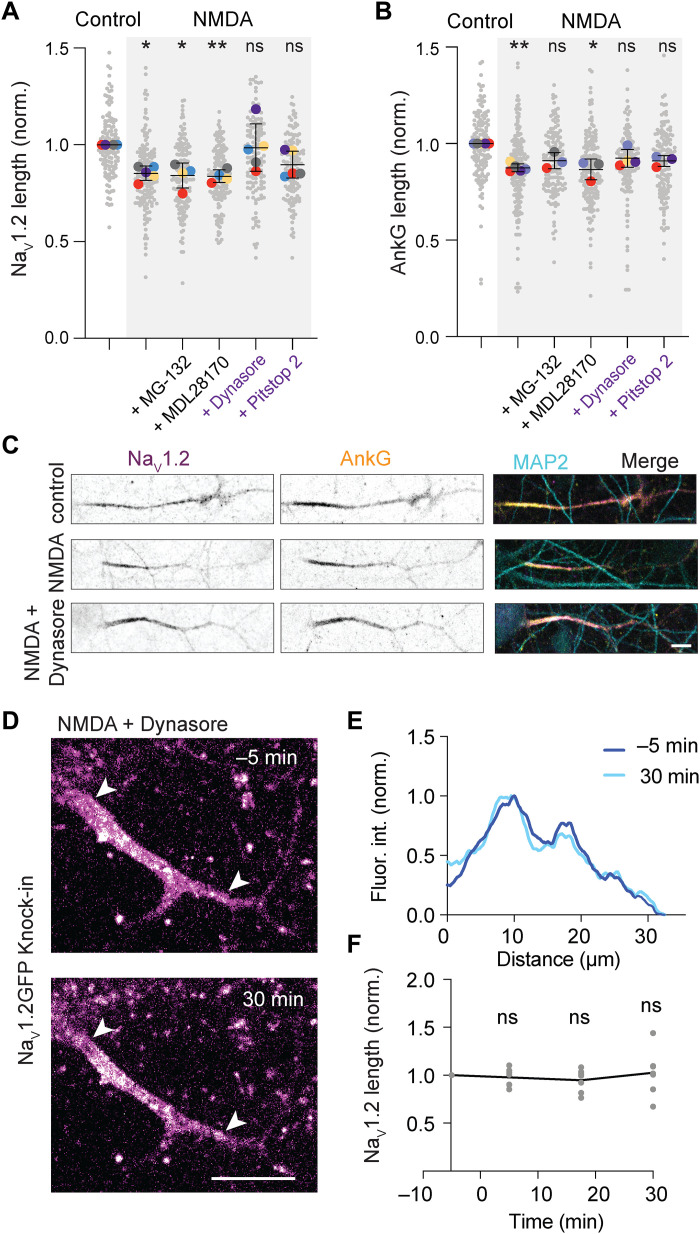
Endocytosis is required for NMDA-induced Na_V_1.2 removal from the AIS. (**A**) Na_V_1.2 and (**B**) AnkG relative length following NMDA application and in the presence of MG-132, MDL2870, Dynasore, and Pitstop 2, *N* = 3 to 6 independent experiments and *n* > 100 neurons per condition per experiment. Kruskal-Wallis test; NMDA: Na_V_1.2, *P = 0.019; AnkG, **P = 0.001; NMDA + MG-132: Na_V_1.2, *P = 0.036; AnkG, *P* = 0.21 (ns); NMDA + MDL28170: Na_V_1.2, **P = 0.005; AnkG, *P = 0.018; NMDA + Dynasore: Na_V_1.2, *P* > 0.99 (ns); AnkG, *P* = 0.38 (ns); NMDA + Pitstop 2: Na_V_1.2, *P* = 0.068 (ns); AnkG, *P* = 0.39 (ns). (**C**) Immunostaining for Na_V_1.2, AnkG, and MAP2 of DIV14 neurons in control conditions and NMDA with or without the dynamin inhibitor Dynasore. Scale bar, 10 μm. (**D**) Live-cell imaging of Na_V_1.2-GFP knock-in neuron 5 min before and 30 min after NMDA treatment and control. Scale bar, 10 μm; white arrowheads point to the start and end point of the Na_V_1.2-GFP signal. (**E**) Corresponding fluorescence intensities normalized to the maximum intensity. (**F**) Average Na_V_1.2-GFP length of neurons treated with NMDA in the presence of Dynasore normalized to the first frame. Repeated-measure one-way ANOVA with Šídák’s multiple comparisons test, *P* > 0.70 (ns), *n* = 7 neurons in *N* = 2 cultures.

How are Na_V_ channels removed from the distal AIS during NMDAR-mediated AIS plasticity? To answer this question, we pharmacologically inhibited common signaling pathways known to promote protein removal and/or degradation (Fig. 4, A and B, and fig. S6). We first tested the role of calpains, a family of calcium-dependent proteases. Although calpains have been shown to be involved in stress-mediated AIS disassembly (*10*, *11*, *40*–*42*), inhibition with the selective calpain inhibitor MDL 28170 during induction of NMDAR-mediated plasticity neither blocked the ~15% shortening of Na_V_1.2 nor AnkG (Fig. 4, A and B). Since it was shown previously that AIS proteostasis can be modulated by local Ecm29-targeted proteasome (*43*), we then tested the role of proteasome-mediated degradation. Application of NMDA in the presence of the proteasome-blocker MG-132 prevented the shortening of AnkG, but not Na_V_1.2 (Fig. 4, A and B, and fig. S6A). Last, we addressed the involvement of endocytosis in controlling Na_V_1.2 redistribution during plasticity. Na_V_1.2 and Na_V_1.6 channels have been reported to contain endocytic motifs, which are important for their stabilization at the AIS membrane (*25*, *44*). Consistent with these observations, the application of two distinct blockers for endocytosis, either by inhibiting dynamin guanosine triphosphatase (GTPase) activity (Dynasore, 80 μM) or clathrin (Pitstop 2, 20 μM), abolished the NMDA-induced shortening of Na_V_1.2 and AnkG (Fig. 4, A to C, and fig. S6, A and B). To further corroborate this result in live neurons, we imaged individual Na_V_1.2-GFP knock-in neurons before and after the application of NMDA in the presence of Dynasore. Na_V_1.2 length remained unchanged, confirming that blocking dynamin prevents NMDA-induced AIS plasticity (Fig. 4, E and F). We verified that none of these drugs alone affect AnkG nor Na_V_1.2 lengths at the population level (fig. S6B) and that incubation with Dynasore alone during live imaging did not trigger AIS lengthening (fig. S6C). Together, our results show that endocytosis is required for NMDAR-dependent AIS plasticity.

### Endocytic structures are recruited at the AIS during rapid plasticity

**Fig. 5.**
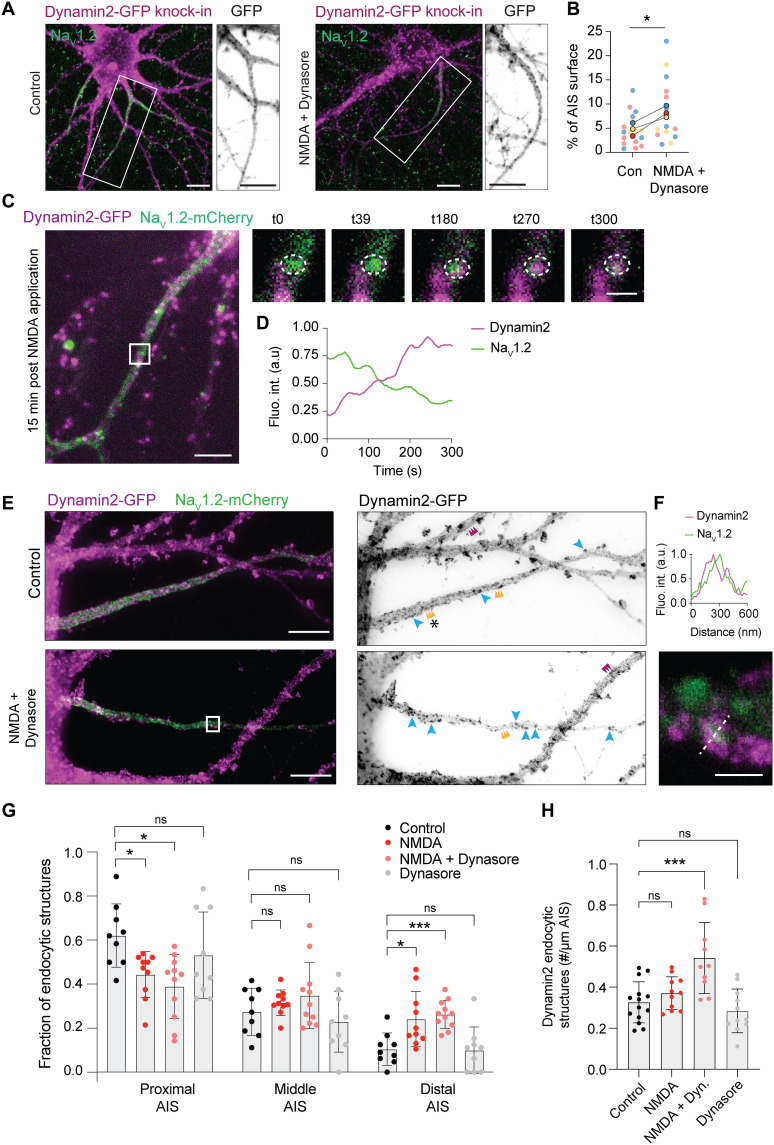
Recruitment of the endocytic machinery at the AIS during rapid plasticity. (**A**) Dynamin2 GFP knock-in neurons stained for GFP reveals increased NMDA-induced clusters in the AIS. Scale bars, 10 μm. (**B**) Dynamin2 surface in the Na_V_1.2-positive AIS in indicated conditions. Paired *t* test, *P = 0.033. *N* = 3 independent experiments, four to seven neurons per condition per experiment. (**C**) Simultaneous dual-color live imaging of a neuron coexpressing dynamin2-GFP and Na_V_1.2-mCherry 15-min post-NMDA treatment. Time points (in seconds) of the boxed areas are presented. Scale bars, 5 μm (left) and 500 nm (zooms). (**D**) Average fluorescence intensity inside the circle over time, smoothened over 24 s. (**E**) STED images of neurons coexpressing dynamin2-GFP and Na_V_1.2-mCherry under indicated conditions. Scale bars, 5 μm. Blue arrowheads point to endocytic structures, colored combs show regions with periodic distribution of dynamin2 in the AIS (orange) or dendrites (magenta). (**F**) Zoom of the boxed area and fluorescence intensity profile along the dashed line. Scale bar, 250 nm. (**G**) Distribution of dynamin2 endocytic structures along the AIS in indicated conditions. Two-way ANOVA with Tukey’s multiple comparison test. Proximal AIS: control versus NMDA, *P = 0.015; control versus NMDA + Dynasore, *P = 0.037; control versus Dynasore, *P* = 0.69 (ns). Middle AIS: control versus NMDA, *P* = 0.58 (ns); control versus NMDA + Dynasore, *P* = 0.73 (ns); control versus Dynasore, *P* = 0.86 (ns). Distal AIS: control versus NMDA, ***P = 0.0006; control versus NMDA + Dynasore, *P = 0.047; control versus Dynasore, *P* = 0.99 (ns); *n* > 9 neurons from two independent experiments. (**H**) AIS density of dynamin2 structures under indicated conditions. One-way ANOVA with Dunnett’s multiple comparison test. Control versus NMDA, *P* = 0.68 (ns); control versus NMDA + Dynasore, ***P = 0.0002; control versus Dynasore, *P* = 0.72 (ns); *n* > 9 neurons from two independent experiments.

The reduction of Na_V_1.2 channels during plasticity could be mediated by local endocytosis at the AIS or by lateral diffusion before internalization. To address the involvement of endocytosis during AIS plasticity, we first assessed the presence at the AIS of two members of the endocytic machinery, dynamin2 and clathrin light chain, using CRISPR-Cas9 GFP knock-ins (*28*). We detected GFP puncta throughout the transfected neurons including at the AIS, stained with Na_V_1.2 (Fig. 5A and fig. S7A). To visualize the putative internalization of Na_V_1.2 channels after NMDA treatment, we performed live imaging of neurons overexpressing dynamin2-GFP and Na_V_1.2-mCherry. We first verified that overexpressed channels behave as endogenous channels in response to NMDA treatment. Live imaging of Na_V_1.2-GFP showed that GFP length remained constant under control conditions, while it significantly decreased 30 min after NMDA application (fig. S7, D and E and cf. Fig. 2). Dual-color live imaging of Na_V_1.2-mCherry and dynamin2-GFP at various time points after NMDA application allowed us to visualize a few events at the AIS where dyamin2-GFP progressively localized to circular structures of Na_V_1.2-mCherry, while Na_V_1.2 signal progressively disappeared (Fig. 5, C and D). However, as endocytosis is a fast process and happens at discrete locations, it was challenging to catch these events. We therefore decided to quantify the recruitment of dynamin-positive endocytic structures at the AIS following NMDA application in the presence of Dynasore. Dynasore inhibits the GTPase activity of dynamin thus only preventing the fission of endocytic pits but does not block the initiation of new clathrin-coated pits (*45*, *46*). Applying Dynasore during and after NMDA application allowed us to visualize endocytic structures, which formed during AIS plasticity. We observed a significant increase of the relative surface area covered by dynamin2-GFP at the AIS (~1.5-fold) and of the average cluster area under the NMDA condition (~1.5-fold) in the presence of Dynasore (Fig. 5, A and B, and fig. S7C). No change was detected in the pattern of clathrin light chain (fig. S7B). To characterize the dynamin2 structures with higher spatial resolution, we performed STED imaging on neurons coexpressing dynamin2-GFP and Na_V_1.2-mCherry. At the nanoscale, dynamin2 organized in two different patterns. Dynamin2 showed a periodic distribution in numerous regions of the axon and dendrites of control neurons (Fig. 5E, combs, and fig. S7F). Although it was less regularly organized than actin and spectrins (*47*, *48*), the analysis of the autocorrelation profile of dynamin2 fluorescence intensity revealed a periodicity of ~230 nm (fig. S7, F and G). This indicates that dynamin2 might locally be part of the axonal submembrane cytoskeleton. Moreover, dynamin2 also formed circular, O-shaped structures (Fig. 5E, blue arrowheads) of around ~220 nm of diameter (fig. S7H), resembling typical endocytic structures reported for clathrin and dynamin in dendrites of neurons and other cell types (*45*, *46*, *49*). We then characterized the distribution of dynamin2 endocytic structures along the AIS under various conditions (Fig. 5G). Control neurons showed a high proportion of endocytic structures in the proximal part of the AIS (~60%; Fig. 5G). Notably, application of NMDA caused a notable relocation by significantly increasing the fraction of endocytic structures in the distal AIS. This increase was also observed after NMDA treatment in the presence of Dynasore, but not following Dynasore treatment alone (Fig. 5G), suggesting that this change is associated with NMDAR-mediated AIS plasticity. Moreover, we detected a significant increase in the number of dynamin2 endocytic structures under the NMDA + Dynasore condition, consistent with a recruitment of the endocytic machinery at the AIS during rapid plasticity (Fig. 5H). Under this condition, we were also able to detect Na_V_1.2-mCherry signal within dynamin2 endocytic structures (Fig. 5F), further supporting the hypothesis that Na_V_1.2 channels can be endocytosed at the AIS during plasticity.

Together, our data show that activity-dependent Na_V_1.2 channel removal from the AIS membrane is driven by clathrin-mediated endocytosis. Our results further suggest that a pool of dynamin2-positive endocytic structures is recruited to and retained at the distal AIS during the process of activity-dependent plasticity. Moreover, these dynamin2 endocytic structures were associated with Na_V_1.2 channels. While we do not exclude that Na_V_1.2 channels could diffuse out of the AIS before being internalized, our data show that those channels can be endocytosed at the AIS during rapid plasticity.

### Endogenous Na_V_1.2 displays a higher mobility in the distal AIS

Since dynamin2-positive endocytic structures are distributed uniformly along the AIS following NMDA application, we wondered why the distal pool of Na_V_1.2 channel is preferentially affected during plasticity. Previous studies show that the interaction of Na_V_1 channels or neurofascin-186 with the scaffold AnkG limits their membrane diffusion and locally prevents their internalization (*22*, *24*, *44*, *50*). Therefore, we assessed whether local differences in Na_V_1.2 anchoring under basal conditions could explain its preferential loss from the AIS distal region during plasticity. We first performed a detergent extraction experiment on live neurons to address the tethering of Na_V_ channels to the submembrane cytoskeleton (fig. S8, A to C) (*51*). After extraction, we observed significantly shorter AIS when stained for AnkG (16 ± 1.2% reduction) and Na_V_1.2 (26 ± 2.2%) and Na_V_1.6 (17 ± 1.6%; fig. S8, A and B). The analysis of Na_V_1.2 and Na_V_1.6 relative expression patterns revealed a large loss of Na_V_1.2 after extraction specifically in the distal AIS (fig. S8C), indicating that the anchoring of this channel isoform is weaker in this subcellular region. To test this with an alternative method, we performed fluorescence recovery after photobleaching (FRAP) experiments to measure the mobility of endogenously tagged Na_V_1.2-GFP in DIV14 neurons (fig. S8, D and E). We bleached separate regions within the AIS to compare the recovery in the proximal versus the distal part. The results showed a significantly larger recovery in the distal AIS (~10% average recovery after 45 min) compared to the proximal region (~7%; fig. S8, F and G). The fluorescence recovered from the distal axon to the AIS (fig. S8E, white arrows), suggesting that Na_V_1.2 channel accumulation at the AIS arises from retrograde diffusion along the axon as previously reported for neurofascin-186 and K_V_7.3 (*22*). Comparisons of distal versus proximal recovery percentage for multiple experiments confirmed a significant ~1.5-fold higher mobility of Na_V_1.2 in the distal AIS (fig. S8G), further highlighting local differences in Na_V_1.2 anchoring properties. The reduced anchoring of Na_V_1.2 channels in the distal AIS could thus explain why this isoform is lost in the distal AIS during plasticity.

### NMDAR-mediated AIS length reductions increase the AP threshold

**Fig. 6.**
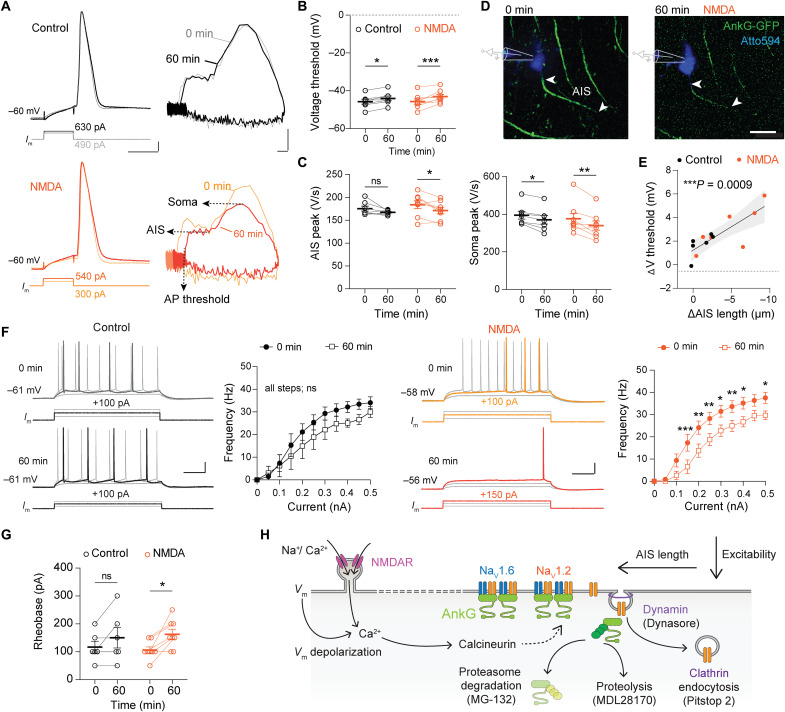
NMDA-induced AIS shortening correlates with increased threshold for AP generation. (**A**) AP voltage-time and phase-plane plots before and after NMDA and APV. Scale bars, 3 ms, 10 mV and 10 mV, 100 V/s. (**B**) Voltage threshold significantly increased after 60 min [two-way ANOVA, *P* = 0.0001 (time), *P* = 0.78 (treatment), and *P* = 0.278 (interaction). Šídák’s multiple comparisons test: Ctrl, *P < 0.05; NMDA, ***P < 0.001]. (**C**) NMDA reduces AP rate of rise. AIS peak: two-way ANOVA, *P* = 0.01 (time), *P* = 0.49 (treatment), and *P* = 0.54 (interaction). Multiple comparisons tests: Ctrl, *P* = 0.29 (ns); NMDA, *P = 0.047. Soma peak: two-way ANOVA, *P* = 0.0005 (time), *P* = 0.48 (treatment), and *P* = 0.40 (interaction). Multiple comparisons test: Ctrl, *P = 0.045; NMDA, **P = 0.0028. (**D**) Live AnkG-GFP imaging during NMDA treatment. See also movies S1 and S2. Scale bar, 20 μm. (**E**) AIS length reduction significantly correlates with voltage threshold. Continuous line, linear regression with 95% confidence interval (shaded). (**F**) Current-frequency (*I*-*f*) relationships for control (black) and NMDA treatment (orange). Control: paired mixed-effects model, *P* < 0.0001 (current), *P* < 0.0003 (time), and *P* = 0.69 (interaction). Individual current injections were not different (Šídák’s multiple comparisons test, *P* > 0.05 for all steps tested). NMDA reduced excitability. Two-way ANOVA, *P* < 0.0001 (current), *P* < 0.0001 (time), and *P* = 0.046 (interaction). Multiple comparisons tests for current steps; *P < 0.05, **P < 0.01, and ***P < 0.001. Scale bars, 100 ms, 20 mV. (**G**) Rheobase current increased after NMDA. Two-way ANOVA, *P* = 0.97 (treatment), *P* < 0.007 (time), and *P* = 0.41 (interaction). Multiple comparisons tests: Ctrl, *P* = 0.25 (ns); NMDA, *P = 0.017. (**H**) Proposed AIS plasticity model (see Discussion). (A to G) Data are depicted as individual scatter and mean ± SEM.

Dynamic changes in distal Na_V_1.2 channels are expected to be accompanied by a shift in the intrinsic neuronal excitability since the distal AIS plays a critical role in the initiation of APs (*1*, *52*). To test this, we recorded in acute slices from the AnkG-GFP mice and recorded the AP properties in CA1 pyramidal neurons in whole-cell configuration and simultaneously imaged the AIS (every 5 min; Fig. 6 and movies S1 and S2). In these experiments, the resting membrane potential remained constant for 1 hour [paired two-way analysis of variance (ANOVA), *P* = 0.052 for time, *P* = 0.73 treatment, *n* = 15; fig. S9]. Also, the current threshold was unaffected (two-way ANOVA, *P* > 0.05). However, in both control and the NMDA-treated neurons, the voltage threshold for initiating an AP significantly increased (Fig. 6, A and B). The maximum peak in the AP rate of rise, reflecting the somato-dendritic Na_V_ activation, was reduced in both control and NMDA groups, suggesting that during these recordings also NMDA-independent changes occurred (Fig. 6C). However, the AIS component of the AP, reflecting the antidromic invasion and determining the AP onset, was selectively and significantly reduced by NMDA application (~13 V/s reduction; Fig. 6C). To examine the contribution of the geometrical AIS length changes, we leveraged the simultaneous live imaging of AnkG-GFP with the recording from the same neurons and plotted the AIS length change against the properties of the changes in AP (Fig. 6, D and E). Notably, a linear regression of AIS length change revealed a significant and strong prediction of the AP voltage threshold change (~0.5 mV/μm, *r*^2^ = 0.065, *P* = 0.0009, *n* = 13; Fig. 6E). The NMDAR-induced AP voltage threshold change also temporally correlated with the AIS length change (fig. S9). Furthermore, NMDA treatment, but not control treatment, significantly reduced the firing probability during long-duration current injections (Fig. 6F). Last, the NMDA-specific reduction in excitability was also reflected in an increased rheobase to evoke AP trains with long duration pulses (Fig. 6G).

Together, these data suggest a model of rapid AIS plasticity in which synaptic NMDAR-mediated Ca^2+^ influx, together with local AIS Ca^2+^ and calcineurin activation, causes a dynamin-clathrin–dependent endocytosis of axonal Na_V_1.2 channels, functionally reducing the input-output transformation (Fig. 6H).

## DISCUSSION

Endogenous labeling of Na_V_ channels and AnkG [this study and (*27*, *31*)] allowed us to longitudinally image, without overexpression artifacts, central AIS proteins during activity-dependent plasticity. These tools enabled interrogation and identification of molecular mechanisms mediating AIS remodeling at high spatiotemporal resolution at a single-cell level while avoiding the variability of cell population analysis. Within minutes, the brief application of NMDA triggered structural AIS plasticity predominantly characterized by the loss of AnkG and Na_V_1.2 channels along several micrometers of the distal AIS (Fig. 2). The signaling pathway was initiated by synaptic NMDARs and involved PPs such as calcineurin and ultimately activated clathrin-mediated endocytosis (Figs. 3 to 5). Last, we found that dynamin2 was recruited at the distal AIS during the rapid remodeling of this region (model in Fig. 6H). Live imaging provided structure-function assessment within the same neuron showing that AIS shortening at the micrometer scale predicts the changes in threshold behavior of the AP (Fig. 6E). Notably, the rapid disassembly of the distal AIS was not directly dependent on synaptic depression, since inducing LTD electrically or with DHPG was insufficient to trigger AnkG shortening. However, NMDAR-induced LTD and AIS shortening did evolve on the same time scale and shared multiple molecular mechanisms, from the role of clathrin-dependent endocytosis in the removal of AMPARs from the PSD (*53*, *54*) to the requirement of calcineurin-mediated dephosphorylation (*55*, *56*). In future experiments, it would be interesting to investigate whether calcineurin directly dephosphorylates Na_V_1.2 and address how this affects Na_V_1.2 binding to AnkG and membrane dynamics of the channel. Na_V_1.2 bears an endocytic motif in the II-III loop, which is masked by the interaction with AnkG and thereby could block the internalization of the channel at the AIS (*44*). Interaction with AnkG alone, however, might not be sufficient to prevent internalization, as shown for the MAP1B-binding mutant Na_V_1.6 channel, which is still capable to bind to AnkG but accumulates less at the AIS membrane because it undergoes endocytosis (*25*, *57*). Another mechanism that AIS shortening shares with synaptic LTD is the contribution of the ubiquitin proteasome degradation system (UPS), which controls PSD-95 and AKAP150 levels during LTD (*58*, *59*). Similarly, we observed that blocking the UPS prevents the shortening of AnkG following NMDA treatment (Fig. 5B), indicating that AIS remodeling could also rely on protein degradation. A role for the UPS in the AIS proteostasis, as well as for 190-kDa AnkG stability at the spine, has previously been reported (*43*, *60*). If AnkG is targeted by the UPS, then this is not the case for Na_V_1.2 (Fig. 4, B and C), and in future studies, it would be important to assess the fate of endocytosed Na_V_1.2 channels. Endocytosed Na_V_1.2 channels could be targeted to lysosomal degradation or may undergo local recycling, which could provide a “readily-releasable” recycling pool of Na_V_ channels, and it is tempting to speculate that such a pool could be used during rapid AIS remodeling. Once endocytosed, the targeting of Na_V_1.2 for recycling versus degradation might rely on the duration and concentration of cytoplasmic Ca^2+^. In the case of excitotoxic events, the AIS is disrupted in an irreversible manner. Applying high concentration of glutamate causes the endocytosis and subsequent degradation of AIS K_V_7.2/7.3 channels, in an NMDAR-dependent manner (*10*). A recent study reports a role for local AIS clathrin-mediated endocytosis of somato-dendritic receptors in the maintenance of neuronal polarity (*26*). In this case, endocytosis is mediated by dynamin1 and internalized receptors are targeted for degradation. It would be interesting to investigate what determines the specificity of the cargos being internalized during basal or activity-driven endocytosis. Here, we observed a change in the distribution of dynamin2 endocytic structures along the AIS during plasticity (Fig. 5). Under basal conditions, most of these structures localized to the proximal AIS and we therefore hypothesize that somato-dendritic proteins might be preferentially endocytosed from the proximal AIS (*26*), while AIS plasticity triggers the recruitment of dynamin2 to the distal part of the AIS, inducing internalization of Na_V_1.2. Moreover, different sets of adaptor proteins might be involved in basal versus activity-driven endocytosis. The adaptor complex protein AP-2 localizes at the AIS and has been implicated in basal endocytosis of somato-dendritic receptors at the AIS (*26*), but it also interacts with Na_V_1.2 channels (*57*), leaving it open to determine whether other signals might be involved.

Our results suggest that for rapid AIS shortening temporal coincidence of synaptic activation and postsynaptic depolarization is required: The length of AnkG-GFP along the AIS remained stable with low-frequency presynaptic stimulation inducing e-LTD, as well as during somatically evoked postsynaptic depolarization with AP firing (fig. S5). Such temporal coincidence was experimentally realized by chemical application of the agonist NMDA [globally depolarizing dendritic branches and the axo-somatic membrane potential causing AP generation (this study and (*29*)]. Whether physiologically relevant stimuli induce a coincidence detection at the AIS with local ion channel endocytosis/insertion remains, however, to be further investigated. Direct experimental evidence would require monitoring AnkG-GFP while triggering presynaptic inputs with postsynaptic depolarization in a temporally controlled manner, as for example with spike timing–dependent plasticity experiments. Rapid AIS plasticity evoked by correlated synaptic activation and AP generation may provide a mechanistic explanation for the synergistic scaling of the AP threshold and synaptic responses during NMDAR-dependent long-term potentiation (*38*). Furthermore, our findings raise the intriguing possibility that dendritic NMDA spikes evoke AIS plasticity. These large and prolonged plateau depolarizations, occurring in vivo during sensory integration, amplify dendritic Ca^2+^ influx and are key integrating signals for synaptic plasticity (*61*, *62*). Our data are not excluding activity-dependent Na_V_ channel plasticity occurring elsewhere along the somato-dendritic membrane or the expression of NMDA-independent excitability changes. In our experiments, we also observed changes in the somato-dendritic component of the AP, suggesting a reduction of somatic Na_V_ channels, in line with previous work (*38*). Activity-dependent AIS plasticity could be further studied by combining electrophysiological recordings with Dynasore to test the role of endocytosis more directly. The AnkG-GFP transgenic mouse and Na_V_ live markers [this study and (*31*)] provide exciting avenues to experimentally correlate cytoplasmic Ca^2+^ imaging with ion channel membrane dynamics and establish how spatiotemporal patterns of intracellular Ca^2+^ are linked with AIS Na_V_1.2 membrane endocytosis and insertion. Spike threshold changes are a hallmark of intrinsic plasticity and observed across many species and learning paradigms (*63*). Since activity-dependent changes of the AIS provide a critical hub for short- and long-term intrinsic plasticity, cooperation with synaptic plasticity via common molecular pathways may represent a powerful mechanism to change the neuronal ensemble activity and engram formation during learning and memory.

## MATERIALS AND METHODS

### Animals and ethics

Double-floxed AnkG-GFP mice [B6;129SV-ank3tm1DCI/HD (*27*)] of both sexes were kept at a 12-hour light-dark cycle (lights on at 07:00, lights off at 19:00) with ad libitum food and water. All animal experiments were performed in compliance with the European Communities Council Directive 2010/63/EU effective from 1 January 2013 and with Dutch national law (Wet op de Dierproeven, 1996). The experiments and ethics were evaluated and approved by the national committee of animal experiments application numbers AVD 80100 2017 2426 and AVD 80100 2022 16329 [Royal Netherlands Academy of Arts and Sciences (KNAW)] and AVD 10800 2017 3404 (Utrecht University). The animal experimental protocols were designed to minimize suffering, approved and monitored by the KNAW animal welfare body (NIN 20.21.07).

### AAV injections

Between 4 to 6 weeks of age, mice were anesthetized with isoflurane and head-fixed in a stereotactic apparatus (Kopf Instruments). Body temperature was kept at 35°C with a heating pad. A glass pipette was inserted bilaterally through a small craniotomy (stereotactic coordinates: 3.0 mm P, 3.5 mm L, and 3.0 to 1.5 mm depth). Fifty nanoliters of undiluted pENN.AAV.CamKII 0.4.Cre.SV40 (plasmid no. 105558, AAV5, Addgene) was injected into the CA1 area of the hippocampus. Cre-recombinase flips the orientation of a cassette containing both the *Ank3* last exon and the coding sequence of eGFP, thus inducing expression of Cre-dependent AnkG-GFP fusion protein. Afterward, the skin was closed with sutures and mice received meloxicam [1 mg/kg body weight (BW); Metacam, Boehringer-Ingelheim, Germany; subcutaneously as postoperative pain medication]. Mice were allowed to recover for at least 2 weeks before euthanasia, to allow for sufficient virus expression.

### Acute slice preparation

Between 6 and 8 weeks of age, mice were euthanized for preparation of acute hippocampal brain slices. Mice were deeply anesthetized by application of pentobarbital i.p. (60 mg/kg BW). They were perfused with ice-cold oxygenated cutting artificial cerebrospinal fluid (ACSF; consisting of 125 mM NaCl, 25 mM NaHCO_3_, 1.25 mM NaH_2_PO_4_, 3 mM KCl, 25 mM glucose, 1 mM CaCl_2_, 6 mM MgCl_2_, and 1 mM kynurenic acid, saturated with 95% O_2_ and 5% CO_2_, pH 7.4) and subsequently decapitated. The brain was swiftly removed and submerged in ice-cold cutting ACSF (see above for composition). The 300-μm-thick transverse acute hippocampal slices were cut using a Leica VT 1200S vibratome (Leica Biosystems, Wetzlar Germany). Slices were allowed to recover for at least 35 min (60 min for e-LTD experiments) at 37°C and subsequently kept in a holding chamber at room temperature (RT) until experiments commenced. Recordings and incubation experiments were carried out at ~32°C.

### Acute slice plasticity experiments

After sectioning and recovery, the slices were gently transferred onto cell culture inserts (Millicell, 30 mm, Merck) in a small petri dish that was constantly perfused with ACSF (125 mM NaCl, 25 mM NaHCO_3_, 1.25 mM NaH_2_PO_4_, 3 mM KCl, 25 mM glucose, 2 mM CaCl_2_, and 1.3 mM MgCl_2_ saturated with 95% O_2_ and 5% CO_2_, pH 7.4) at 6 ml/min. After transfer, the chamber was perfused with 20 μM NMDA (Sigma-Aldrich) in ACSF for 3 min, directly followed by perfusion with 100 μM APV (Alfa Aesar) for 3 min to prevent prolonged NMDAR opening. Control treatment solely consisted of perfusion of slices with 100 μM APV for 3 min. Subsequently, slices were fixated using 4% paraformaldehyde [PFA; in 0.1 M phosphate-buffered saline (PBS)] for 20 min at two time points: before perfusion and after 30-min post-treatment. NMDA-treated slices and control slices were taken from corresponding hemispheres at matching CA1 locations (dorsoventral axis) to exclude potential effects of location on AIS length. Slices were then subjected to multichannel immunofluorescent staining. Slices were incubated in blocking buffer [10% normal goat serum (NGS), 1% Triton X-100 in 0.1 M PBS] for a minimum of 90 min at RT, followed by incubation in primary antibodies overnight (5% NGS, 1% Triton X-100 in 0.1 M PBS). Primary and secondary antibodies and concentrations were the same as used for cryosections. Following washing, the slices were incubated with secondary antibodies for a minimum of 4 hours in the dark. Streptavidin Alexa-633 conjugate (1:1000; Invitrogen) was added for the detection of biocytin-filled neurons. Subsequently, slices were washed and mounted using fluorescence-preserving mounting medium (Vectashield, Vector Laboratories). Confocal images of the AIS were acquired using a Leica SP8 confocal microscope (Leica Microsystems, Wetzlar Germany). A 40× objective [oil immersion; numerical aperture (NA), 1.4] was used for population analysis of AIS length. To ensure that the entire AIS structure was in focus, multiple images in the *z*-dimension were projected to one image (maximum intensity projection). Each image section was spaced at 0.5 μm at a total depth of up to 25 μm. Images were taken at 1024 × 1024 resolution, resulting in resolution of 0.27 μm/pixel. Individual AIS length was determined using the morphometrical software analysis tool AISuite (https://zenodo.org/record/4264831). Segments were individually traced, and the threshold determining start and end of each individual segment was set to be 35% of the maximum fluorescent signal of a single AIS.

### Electrophysiology and live AIS imaging

Slices were transferred to an upright microscope (BX61WI, Olympus Nederland BV) and constantly perfused with carbogenated recording ACSF consisting of 125 mM NaCl, 25 mM NaHCO_3_, 1.25 mM NaH_2_PO_4_, 3 mM KCl, and 25 mM glucose. Typically, 2 mM CaCl_2_, 1.3 mM MgCl_2_, and 1 mM sodium ascorbate were added, while 2.5 mM CaCl_2_ and 1 mM MgCl_2_ was used for the e-LTD and depolarization experiments. The chamber was perfused at a rate of 3 ml/min. Neurons were visualized with a 40× water immersion objective (Achroplan; NA, 0.8; infrared 40×/0.80 W, Carl Zeiss Microscopy) with infrared optics and oblique contrast illumination. Patch-clamp recordings were performed from CA1 pyramidal neurons with a GFP-positive AIS identified during a search in epifluorescence mode and visualizing GFP with the 470-nm laser line of a laser diode illuminator (LDI-7, 89 North, USA). Patch pipettes were pulled from borosilicate glass (Harvard Apparatus, Edenbridge, Kent, UK) pulled to an open tip resistance of 4 to 5 megohms and filled with standard intracellular solution containing 130 mM K-gluconate, 10 mM KCl, 10 mM Hepes, 4 mM Mg-ATP, 0.3 mM Na_2_-GTP, and 10 mM Na_2_-phosphocreatine (pH 7.25, ~280 mosmol). For continuous measurements of AP properties over time, we added 1 mM EGTA and 0.1 mM CaCl_2_ to the standard intracellular solution. Atto-594 (20 μM; Atto-tec, Germany) and biocytin (3 mg/ml; Sigma-Aldrich) were routinely added to the intracellular solution to allow for live and post hoc confirmation of colocalization of the AIS with the recorded neuron, respectively. Electrophysiological recordings were performed with an Axopatch 200B (Molecular Devices) or Dagan BVC-700A amplifier (Dagan Corporation, Minneapolis, MN, USA). Signals were analog low-pass filtered at 10 kHz (Bessel) and digitally sampled at 50 kHz using an A-D converter (ITC-18, HEKA Elektronik Dr. Schulze GmbH, Germany) and the data acquisition software Axograph X (v.1.5.4, Axograph Scientific, NSW, Australia). Bridge balance and capacitances were fully compensated in current clamp. Series resistance was compensated to >75% for voltage-clamp recordings. Membrane potentials have not been corrected for the liquid junction potential of the intracellular solution (−14 mV). Once a stable recording was established, AP threshold was recorded at the start (and end) of recordings by injecting 3-ms pulses incrementing 10 pA in amplitude until an AP was generated. In a subset of cells, we continuously generated APs every 5 min, to correlate length changes with electrophysiological changes over time (Fig. 6 and fig. S9). For LTD recordings, an extracellular glass bipolar stimulation electrode was positioned above the SCs, to stimulate presynaptic inputs to CA1. The external electrode was tuned to evoke half of the maximum currents in the recorded cell. Initially, >5 min of baseline was recorded, and all postsynaptic currents were normalized to the mean evoked current amplitude during baseline. For NMDA-induced plasticity experiments, we added 20 μM NMDA (Sigma-Aldrich) for 3 min, similar to previous protocols by (*29*, *64*). Since in acute hippocampal slices, we observed in some cases excessive irreversible depolarization, we subsequently added 100 μM APV (Alfa Aesar) to rapidly antagonize NMDAR-mediated excitation. Control recordings were done with 100 μM APV only. In additional control experiments, we added APV and before, during, and after NMDA (fig. S1, E and F). Neuronal health was further improved by adding sodium ascorbate (1 mM) and increasing MgCl_2_ (1.0 to 1.3 mM) for the NMDA experiments. AnkG-GFP was imaged using an Olympus FV1000 confocal microscope (Olympus Corporation, Tokyo, Japan) controlled by the imaging software FV10-ASW (Ver.03.00). *z*-stacks were acquired at a speed of 12.5 μs/pixel and a resolution of 800 × 800 pixels in 1-μm steps at 1 to 3× zoom. To reduce phototoxicity and minimize bleaching, the *z*-stacks of AIS were taken at four time points (10 min before and at the start of drug exposure, followed by imaging sessions 30 and 60 min afterward). For higher temporal resolution imaging (every 5 min; movies S1 and S2), we used a spinning-disk confocal unit (CSU-X1, Yokogawa) controlled by imaging software Visiview (version 5.0.0.28, Visitron Systems GmbH, Germany). Images were acquired at 2.3 to 9 px/μm resolution and 1 to 4× binning was applied. AIS length was determined with the “Measure ROIs” in Fiji (ImageJ) plugin (https://github.com/cleterrier/Measure_ROIs/blob/56bf5c19c697d1811cc4796588300adeee795c56/Pro_Feat_Fit.js), version from 6 October 2022. Fluorescence intensity thresholds for the detection of AIS beginning and end were adjusted between 10 and 35% depending on the signal-to-noise ratio but kept consistent for an individual time series. For some NMDA (*n* = 12/25) and APV control (*n* = 8/24) experiments, cells were only imaged but not patched. Multiple neurons per slice were imaged simultaneously to increase data yield. In these cases, healthy neurons were selected on the basis of standard visual criteria (low contrast, no swelling or shrinking).

### Primary neuronal cultures and transfection

Primary hippocampal neurons cultures were prepared from embryonic day 18 rat brains (both genders). Cells were plated on coverslips coated with poly-l-lysine (37.5 μg/ml; Sigma-Aldrich) and laminin (1.25 μg/ml; Roche Diagnostics) at a density of 100,000 per well of 12-wells plates. Neurons were first cultured in neurobasal medium (NB) supplemented with 2% B27 (GIBCO), 0.5 mM glutamine (GIBCO), 15.6 μM glutamate (Sigma-Aldrich), and 1% penicillin/streptomycin (GIBCO) at 37°C in 5% CO_2_. From DIV1 onward, half of the medium was refreshed weekly with BrainPhys medium supplemented with 2% NeuroCult SM1 neuronal supplement (STEMCELL Technologies) and 1% penicillin/streptomycin. Hippocampal neurons were transfected using Lipofectamine 2000 (Invitrogen). Briefly, DNA (1.8 μg per well, of a 12-well plate) was mixed with 3.3 μl of Lipofectamine 2000 in 200 μl of NB, incubated for 20 min, and then added to the neurons in neurobasal at 37°C in 5% CO_2_ for 1 hour. Then, neurons were washed with neurobasal and transferred back to their original medium. Transfection of knock-in constructs was performed at DIV3.

### DNA constructs

The knock-in construct for Na_V_1.2 was cloned in the pORANGE (*28*) and the guide RNA used was GGACAAAGGGAAAGATATCA. The GFP tag was added in the C-terminal region (4aa before the STOP codon), mimicking the tagging approach used for Na_V_1.6 (*23*, *65*) or Nav1.2 (*31*), which was shown to not alter channel function nor localization. Na_V_1.2-GFP and Na_V_1.2-mCherry overexpression constructs were generated from a Na_V_1.2-IRES-GFP plasmid shared by K. Bender (*66*). The IRES-GFP fragment was cut out using Xho I–Pac I restriction sites and replaced by GFP or mCherry, amplified by polymerase chain reaction, in frame with Na_V_1.2. Knock-in constructs of GluN1-GFP and clathrin light chain A-GFP (*28*), dynamin2-GFP (*49*), and the overexpression construct of dynamin2-GFP (*67*) have previously been published and shared by H. MacGillavry (University of Utrecht).

### Antibodies

The following antibodies were used in this study: Na_V_1.2 (NeuroMab/Antibodies Incorporated no. 75-024), Na_V_1.6 (Alomone Labs no. ASC-009 and NeuroMab/Antibodies Incorporated no. 73-026), Pan-Nav (Sigma-Aldrich, no. S8809), AnkG (Life Technologies no. 33-8800, Neuromab/Antibodies Incorporated no. 75-146, and Synaptic Systems no. 386-005), βIV-spectrin (Biotrend, provided by M. Engelhardt, Johnnes-Kepler-University, Linz, Austria), MAP2 (Abcam/Bio Connect no. ab5392), GFP (MBL International/Sanbio no. 598 and Abcam nos. ab13970 and ab290), and mCherry (Clontech no. 632543). Corresponding secondary antibodies Alexa-conjugated 405, 488, 568, 594, or 647 goat anti-mouse, anti-mouse immunoglobulin G1 (IgG1)/IgG2a, anti-rabbit, anti–guinea pig, or anti-chicken were used (Life Technologies), as well as streptavidin Alexa-633 conjugate (Life Technologies) for the detection of biocytin-filled neurons. Goat anti-mouse and anti-rabbit Abberior Star580 (no. ST580) and 635P (no. ST635P) were used for STED imaging.

### Pharmacological treatments and plasticity experiments

MG-132 (Calbiochem no. 474790), MDL 28170 (Tocris Bioscience no. 1146/10), MK-801 (Tocris Bioscience no. 924), okadaic acid (Tocris Bioscience no. 1136), tautomycetin (Tocris Bioscience no. 2305), FK-506 (Tocris Bioscience no. 3631), Dynasore (Tocris Bioscience no. 2897), PitStop2 (Abcam no. ab120687), APV (Tocris Bioscience no. 0106), NMDA (Sigma-Aldrich no. 3263), and DHPG (Tocris no. 0805). All chemicals were diluted in a modified Tyrode’s solution (pH 7.4) containing 25 mM Hepes, 119 mM NaCl, 2.4 mM KCl, 2 mM CaCl_2_, 2 mM MgCl_2_, and 30 mM glucose. For NMDA experiments, neurons were first incubated for 5 min in modified Tyrode’s solution, and then NMDA 2× (diluted in modified Tyrode’s at 100 μM) was added to the wells. For controls experiments, Tyrode’s solution was applied twice. After 4 min, neurons were dipped in Tyrode’s solution to wash them and returned to their original medium. NMDA treatment combined with live imaging was performed the same way, and all incubation and washing steps were performed on stage. For DHPG experiment, the same procedure was used as for NMDA-LTD, but neurons were incubated for 5 min in 100 μM DHPG. For Triton X-100 extraction experiment, live DIV14 hippocampal neurons were incubated for 5 min at RT in 0.25% Triton X-100 preheated at 37°C. Neurons were fixed directly after extraction.

### Immunocytochemistry

For immunocytochemistry of cultured neurons, cells were fixed for 6 min with warm PFA (4%)–sucrose (4%). Primary antibodies were incubated overnight at 4°C in GDB buffer (0.2% bovine serum albumin, 0.8 M NaCl, 0.5% Triton X-100, and 30 mM phosphate buffer, pH 7.4). After three washes in PBS, secondary antibodies were incubated in the same buffer for 1 hour at RT. Extracellular staining of GluN1 was performed using a first incubation step with the anti-GFP antibody overnight at 4°C in GDB without Triton X-100, followed by three washes in PBS and incubation with an Alexa-568 secondary antibody in GDB without Triton X-100 for 2 hours at RT. Then, anti-GFP and anti-AnkG antibodies were incubated in GDB buffer containing Triton X-100 overnight at 4°C, washed three times, and corresponding secondary antibodies conjugated with Alexa-405 and Alexa-647 were incubated in the same buffer for 1 hour at RT. Coverslips were mounted using Vectashield (Vectorlabs).

### Immunohistochemistry

To examine the expression of AnkG-GFP mice were deeply anesthetized by application of pentobarbital i.p. (60 mg/kg BW) before transcardial perfusion with PBS for 5 min followed by 4% PFA (in 0.1 M PBS) for 5 min. Brains were postfixed in 4% PFA for 1 hour at RT. After three sucrose cryoprotection steps (1 hour in 10% RT, overnight in 20%, 4°C, overnight in 30% sucrose, 4°C) brains were trimmed to sectioning block in coronal orientation and frozen in embedding medium (Tissue-Tek, Sakura) until further processing. Brains were cut with a cryostat (CM3050 S, Leica) to sections of 40 μm thickness. Slices were incubated in blocking buffer (10% NGS and 0.5% Triton X-100 in 0.1 M PBS) for a minimum of 90 min at RT, followed by incubation in primary antibodies overnight (5% NGS and 0.5% Triton X-100 in 0.1 M PBS). Following washing steps, the slices were incubated with secondary antibodies for a minimum of 90 min in the dark. Subsequently, slices were washed and mounted using fluorescence-preserving mounting medium (Vectashield, Vector Laboratories).

### Image acquisition

Primary neurons were imaged using an LSM700 confocal laser-scanning microscope (Zeiss) with a Plan-Apochromat 63× NA 1.40 oil differential interference contrast (DIC), EC Plan-Neofluar 40× NA1.30 oil DIC, and a Plan-Apochromat 20× NA 0.8 objective. *z*-stacks were acquired with 0.5-μm steps and maximum projections were done from the resulting stack. Acquisition settings were kept the same for all conditions within an experiment.

### gSTED microscopy

Gated STED (gSTED) imaging was performed with a Leica TCS SP8 STED 3× microscope using a HC PL APO 100 × /1.4 oil immersion STED WHITE objective. The 580, 594, 635, and 647 nm wavelengths of pulsed white laser (80 MHz) were used to excite the Abberior Star 580, Alexa-594, Abberior Star 635P, and the Alexa-647 secondary antibodies. All were depleted with the 775-nm pulsed depletion laser. Fluorescence emission was detected using a Leica HyD hybrid detector with a time gate of 0.3 ≤ tg ≤ 6 ns.

### Live-cell imaging and FRAP experiments

Live-cell imaging experiments were performed in an inverted microscope Nikon Eclipse Ti-E equipped with the perfect focus system (Nikon) and a spinning disk–based confocal scanner unit (CSU-X1-A1, Yokogawa). Plan Apo VC 100× NA 1.40 oil, a Plan Apo VC 60× NA 1.40 oil, and a Plan Fluor 40× NA 1.30 oil objectives (all Nikon) were used for acquisition. The system was also equipped with ASI motorized stage with the piezo plate MS-2000-XYZ (ASI), Photometrics PRIME BSI sCMOS camera (Teledyne Photometrics), and controlled by the MetaMorph 7.10 software (Molecular Devices). For imaging, we used 100-mW Vortran Stradus 405 laser (Vortran), 150-mW Vortran Stradus 488 laser (Vortran), 100-mW 561 nm OBIS laser (Coherent), and 150-mW Vortran Stradus 642 nm (Vortran) lasers, a set of ET460/50m, ET525/50m, ET630/75m (red), and ET-EGFP/mCherry filters (Chroma) for spinning disk–based confocal imaging. Optosplit II (Cairn Research) beam splitter was equipped with ET525/50m and ET630/75m emission filters and T585lpxr dichroic mirror (all Chroma). The final resolution using PRIME BSI camera was 0.063 μm/pixel (×100 lens). Coverslips were mounted in metal rings and imaged using a stage top incubator model INUBG2E-ZILCS (Tokai Hit) that maintains temperature and CO_2_ optimal for the cells (37°C and 5% CO_2_). Live imaging was performed in full conditioned medium or in imaging buffer. Time-lapse live-cell imaging of Na_V_1.2 knock-in neurons was performed with a frame rate of 1 acquisition per minute for the FRAP experiments and one acquisition every 7.5 min for the plasticity experiments. FRAP experiments were carried out with an ILas FRAP unit (Roper Scientific France/PICT-IbiSA, Institut Curie). Local photobleaching of Na_v_1.2-GFP in the proximal and distal AIS was performed using regions of interest (ROIs) of 30 × 30 pixels (~25 μm^2^) and recovery was monitored for 45 min, with an image acquisition rate of one image per minute. Dynamin2-GFP and Na_V_1.2-mCherry dual-color imaging was performed with an image acquisition rate of one image per 3 s.

### Image processing and quantification

Movies and images were processed using Fiji (https://imagej.net/software/fiji/) (*68*). Kymographs were generated using the ImageJ plugin from Eugene Katrukha, KymoResliceWide, v0.5 (https://zenodo.org/record/4281086). The AIS position (start and end points), length, and fluorescence intensity of AIS proteins were measured using Christophe Leterrier’s plugin “Pro_Feat_Fit” version from 6 October 2022, (https://github.com/cleterrier/Measure_ROIs/blob/56bf5c19c697d1811cc4796588300adeee795c56/Pro_Feat_Fit.js) and with the following parameters: Small detection, starting point at 50%, and end point at 35% of the max fluorescence intensity. The autocorrelation coefficients were obtained using Christophe Leterrier’s plugin “Autocorrelation” version from 6 October 2022, (https://github.com/cleterrier/Process_Profiles/blob/709147ebae553fe58cc20dc5b630271f64f81d73/Autocorrelation_.js). GluN1, clathrin light chain, and Dynamin2 spots quantifications were performed using the Spot Detector plug-in built-in in Icy software (https://icy.bioimageanalysis.org) (*69*). Quantifications parameters were adjusted per experiment but kept similar for all conditions within each experiment. FRAP recovery was quantified as the percentage of fluorescence recovery depending on the initial fluorescence intensity and taking into account the acquisition photobleaching. A bleaching control was selected outside of the bleached areas and fluorescent signals in the ROIs was set to 100% based on the mean intensity of the first four frames (4 min) before bleaching in the same region and was set to 0% directly after as described previously (*70*). The average recovery rate was calculated by averaging the values of the last five points of fluorescence intensity.

### Statistical analysis

All statistical details of experiments, including the exact values of *n*, and statistical tests performed, are provided in the Results, figures, and figure legends. *n* represents the number of neurons analyzed and *N* the number of independent experiments (or animals). All statistical analyses were performed using Prism9 (GraphPad Software). Significance cutoff was defined at 0.05, with ns as not significant, *P < 0.05, **P < 0.01, and ***P < 0.001. Normality of the data was determined by a D’Agostino and Pearson’s tests. For comparing two groups, parametric two-tailed paired or unpaired *t* tests or nonparametric Mann Whitney or Wilcoxon tests were used. For more than two groups, one- or two-way ANOVA was applied followed by multiple comparisons tests. When values were missing, a mixed-effects analysis followed by multiple comparisons (specific types are indicated in the text) was applied. For all experiments in cultured neurons, the statistical significance was tested on the average values of each independent experiment when at least three independent experiments were performed. For normalized datasets, each value was normalized to the average value of the control condition within the same experiment. Data are plotted as individual values and mean ± SD, unless stated differently in the legend.
