# Supplementary material for: Sodium channel endocytosis drives axon initial segment plasticity
Source: Sci Adv. 2023 Sep 15;9(37):eadf3885. doi: 10.1126/sciadv.adf3885 (PMC10881073; doi:10.1126/sciadv.adf3885)
Supplement: Supplementary file 1 — Figs. S1 to S9 Legends for movies S1 and S2 [file sciadv.adf3885_sm.pdf]

Science Advances
AAAS

Supplementary Materials for

Sodium channel endocytosis drives axon initial segment plasticity

Amélie Fréal *et al.*

Corresponding author: Maarten H. P. Kole, m.kole@nin.knaw.nl; Amélie Fréal, a.freal@vu.nl

Sci. Adv. **9**, eadf3885 (2023)
DOI: 10.1126/sciadv.adf3885

The PDF file includes:

Figs. S1 to S9
Legends for movies S1 and S2

Other Supplementary Material for this manuscript includes the following:

Movies S1 and S2

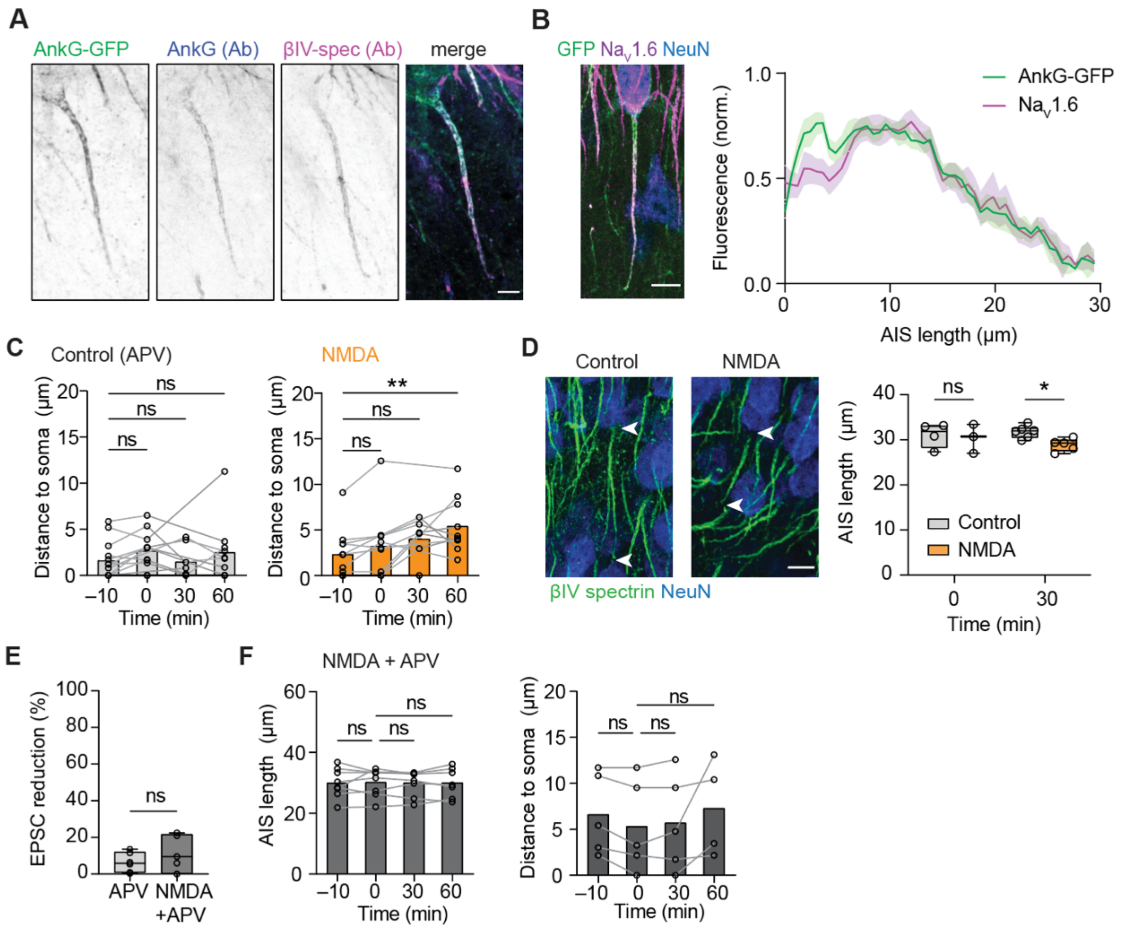

Fig. S1. Validation of AIS targeting of AnkG-GFP and NMDAR-mediated plasticity

(**A**) Example immunofluorescence GFP signal was localized to the AIS and overlapped with AnkG (gp) as well as β4-spectrin (rb) antibody stainings (**B**) Fluorescence intensity distribution shows that $Na_V1.6$ (magenta) overlaps with the AnkG-GFP signals (green) along the AIS (n = 10 AIS). Lines show average (continuous) with SEM (transparent area). (**C**) Analysis of the distance of the AIS onset relative to the soma edge for control (n = 13 neurons, grey) and NMDA (n = 11 neurons, orange). Mixed-effects model (REML) p = 0.455 (control) and *p = 0.0104 (NMDA) for the factor treatment. Dunnett's multiple comparisons test for –10 vs 60 min **p = 0.0095, for all other comparisons p > 0.05 (ns). (**D**) Incubation of acute hippocampal slices in 20 µM NMDA for 3 min followed by 5 min APV (NMDA) vs APV treatment alone (5 min, Control) reveals a significant shortening of the AIS, stained with β4-spectrin, after 30 min recovery. Mixed-effects model *p = 0.017 for the factor treatment. Šídák's multiple comparisons tests ns p = 0.43 (0 min) and *p = 0.017 (30 min). (**E**) Analysis of the EPSC amplitude reduction after perfusion of NMDA in the presence of APV. The amplitude reduction was similar compared to control (Mann-Whitney test, p = 0.5368). (**F**) Constant AIS length and distance to soma in the continuous presence of APV during NMDA application. Mixed-effects model (REML) p = 0.91 (length) and p = 0.18 (distance) for the factor treatment, Dunnett's multiple comparisons for all comparisons p > 0.05 (ns). Scale bars, 5 µm (A, B), 10 µm (D).

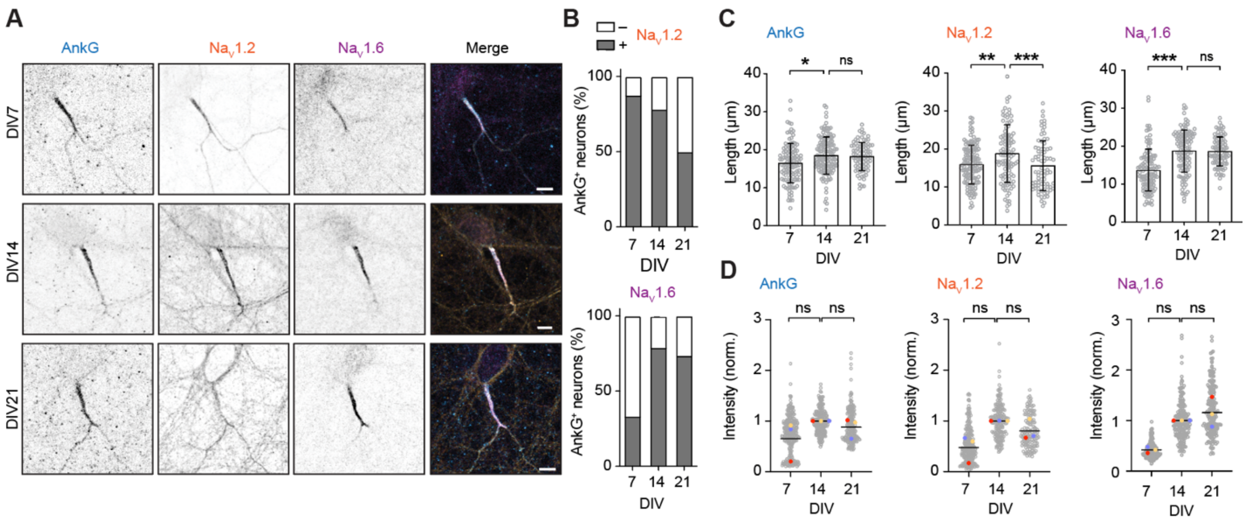

Fig. S2. Hippocampal neuronal AIS expression of Na$_V$1.2 and Na$_V$1.6 during development in culture

(A) Immunostaining of AnkG, Na$_V$1.2 and Na$_V$1.6 in cultured hippocampal neurons at DIV7, 14 and 21. Scale bars, 10 µm. **(B)** Percentage of neurons with AnkG-positive AIS staining positive (grey) or negative (white) for Na$_V$1.2 or Na$_V$1.6. **(C)** Length of AnkG, Na$_V$1.2 or Na$_V$1.6 at DIV7, 14 and 21. Ordinary one-way ANOVA. *n* = 77 to142 neurons (grey) from 3 experiments (colored dots). For AnkG, DIV14 **p = 0.0025, DIV21 ns p = 0.92. For Na$_V$1.2, DIV14 **p = 0.0013, DIV21 ***p = 0.0024. **(D)** Normalized AIS fluorescence intensity of AnkG, Na$_V$1.2 or Na$_V$1.6 staining at DIV7, 14 and 21. Kruskal-Wallis test with Dunn's multiple comparison test, *N* = 3 experiments (22 to 124 neurons per condition per experiment). For AnkG, DIV14 ns p = 0.14, DIV21 ns p > 0.99. For Na$_V$1.2, DIV14 ns p = 0.07, DIV21 ns p > 0.99. For Na$_V$1.6, DIV14 ns p = 0.20, DIV21 ns p > 0.99.

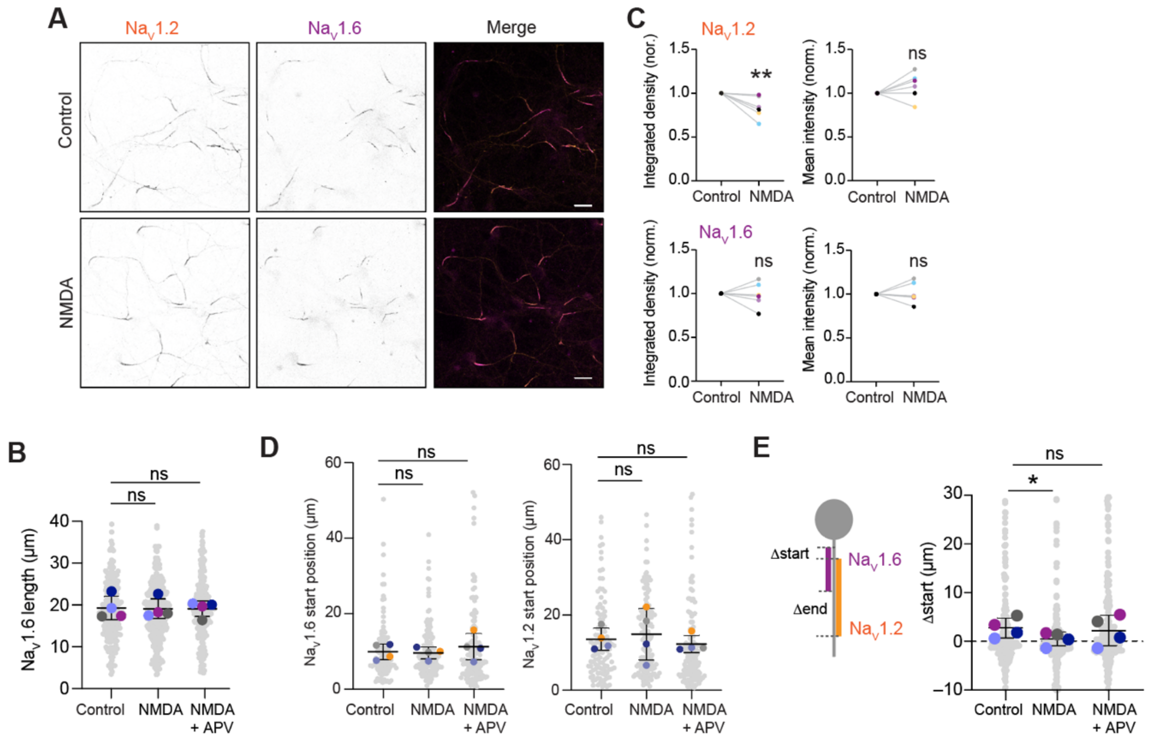

Fig. S3. NMDAR-mediated plasticity affects $Na_V1.2$ but not $Na_V1.6$ membrane expression

(A) DIV14 hippocampal neurons stained for both $Na_V1.2$ and $Na_V1.6$. Scale bars are 30 mm. (**B**) AIS length of $Na_V1.6$ in control, NMDA and NMDA+APV conditions was not affected (Repeated-measure one-way ANOVA with Dunnett's multiple comparisons test, $N = 4$ cultures. Control vs NMDA, ns = 0.93; control vs NMDA + APV, ns p = 0.98, >225 neurons per culture). **(C)** Normalized integrated and mean intensity of $Na_V1.2$ and $Na_V1.6$ in control condition and after NMDA application. Unpaired t-test, $N = 5$ cultures (at least 30 neurons per condition per experiment). For $Na_V1.2$, integrated density **p = 0.009, mean intensity ns p = 0.19. For $Na_V1.6$, integrated density ns p = 0.79, mean intensity ns p = 0.79. (**D**) Absolute start positions of $Na_V1.2$ and $Na_V1.6$ signal onset in control, NMDA and NMDA+APV conditions. Friedman test, $N = 4$ cultures for $Na_V1.2$: NMDA, ns, p = 0.959, NMDA+APV, ns p = 0.959, >108 neurons per condition. For $Na_V1.6$: NMDA, ns, p = 0.577, NMDA+APV, ns p = 0.577, >102 neurons per condition. (**E**) Delta start ($Na_V1.2$ minus $Na_V1.6$ signal onset) along the AIS in control, NMDA and NMDA+APV conditions. RM ANOVA with Dunnett's multiple comparisons test, $N = 4$ cultures. NMDA, *p = 0.048, NMDA+APV, ns p = 0.80, >260 neurons per condition.

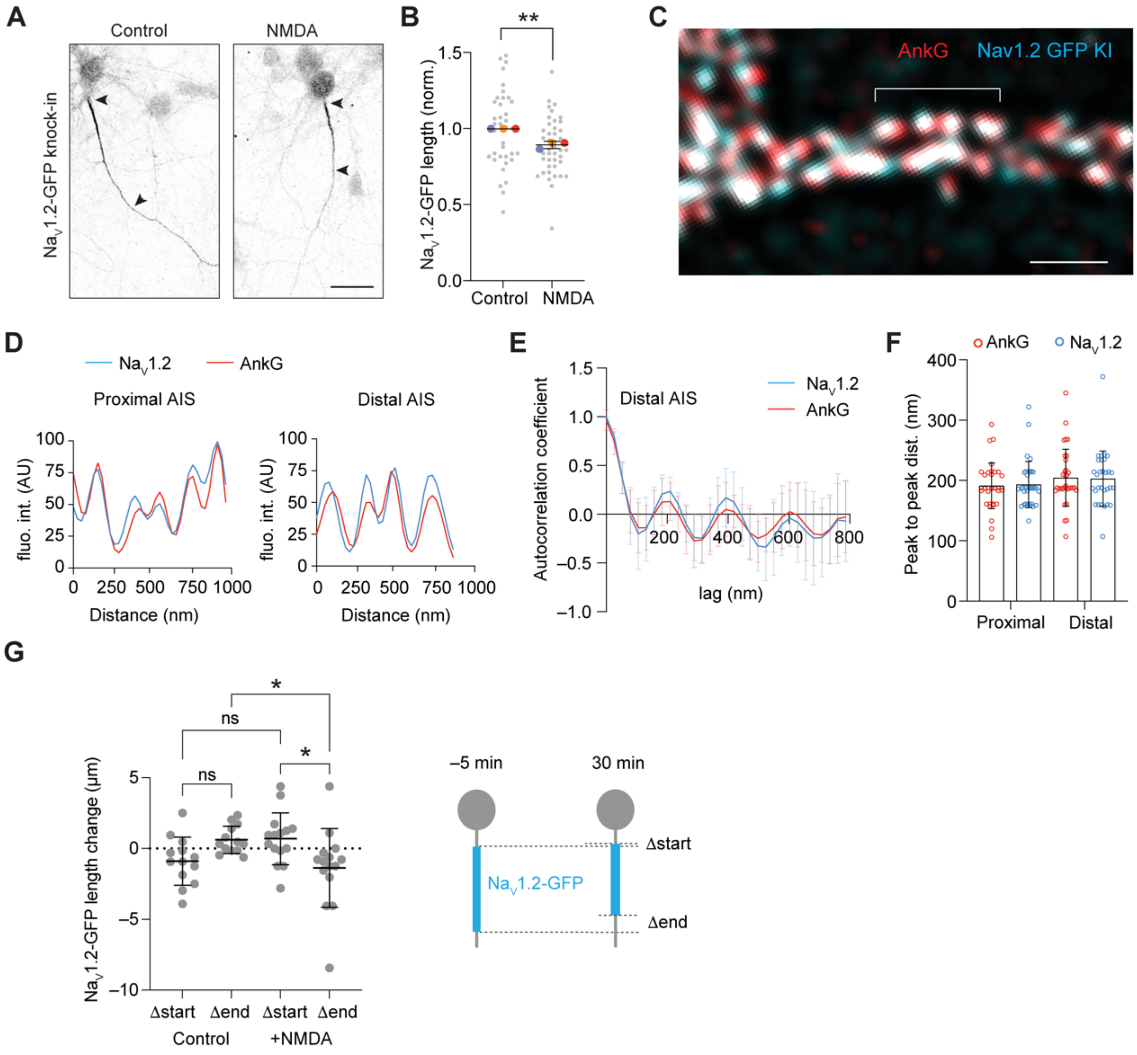

Fig S4. The knock-in of GFP in the C-terminal region of Na$_V$1.2 does not alter the nanoscale organization nor activity-dependent plasticity

(**A,B**) Confocal images of DIV14 Na$_V$1.2-GFP knock-in neurons stained for GFP in control condition and after NMDA application. Arrowheads indicated the start and end position of the AIS. Scale bar, 30 µm. (**B**) Na$_V$1.2-GFP length in NMDA condition relative to the control condition, Unpaired t-test, $N = 3$ independent experiments (colored dots), **p = 0.0019 (n = 48 to 64 neurons, grey dots). **(C)** STED image of the distal portion of the AIS in a DIV14 Na$_V$1.2-GFP knock-in (KI) neuron stained for GFP and AnkG. Scale bar, 500 nm. **(D)** Fluorescence intensity profile of AnkG and Na$_V$1.2 in the proximal and distal AIS, along the brackets shown in **C** and in **Fig. 2F**. (**E**) Autocorrelation profiles of AnkG and Na$_V$1.2 fluorescence intensity in distal AIS regions, n = 8 neurons. (**F**) Mean peak to peak distance of the autocorrelation profiles for AnkG and Na$_V$1.2 in the proximal and distal AIS from n = 30 to 34 measures from 8 neurons. Kruskal-Wallis test followed by a Dunn's multiple comparison test, p > 0.99 for all comparisons. (**G**) Live imaging of NMDAR-mediated Na$_V$1.2-GFP KI changes in the AIS. Kruskal-Wallis test followed by a Dunn's multiple comparison test. Changes in Na$_V$1.2-GFP after 30 min post NMDA application or in control condition. Control, delta start *versus* delta end ns p = 0.08; NMDA, delta start *versus* end *p = 0.024; Delta start control *versus* NMDA ns p = 0.11, Delta end control *versus* NMDA * p= 0.018. Data show scatter and mean ± SD. Control: n = 13 neurons. NMDA: n = 15 neurons.

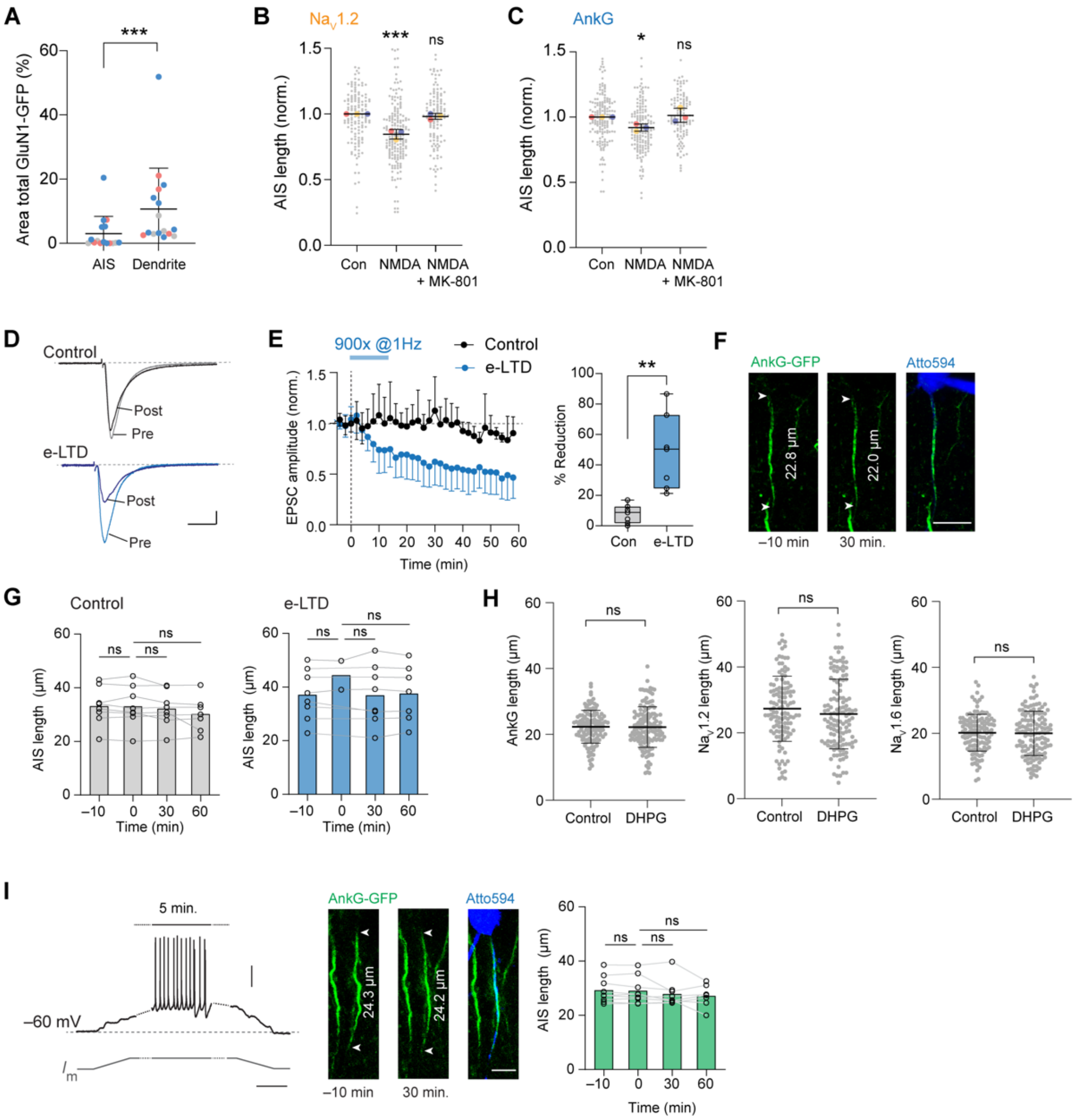

Fig. S5. AIS stability during electrical LTD and action potential firing

(A) Analysis of total GFP staining area in the dendrites and AIS in neurons knock-in for GluN1-GFP. Wilcoxon test, *n* = 16 neurons, from 3 independent experiments ***p < 0.0001. (**B,C**) Length of Na$_V$1.2 (A) and AnkG (B) after NMDA or NMDA+MK-80 relative to control condition. N=3 experiments, Kruskal-Wallis test with Dunn's multiple comparison test. For AnkG NMDA *p = 0.045, NMDA+MK-801 ns p > 0.99. For Na$_V$1.2 NMDA *p = 0.046, NMDA+MK-801 ns p > 0.99. At least 80 neurons per condition per experiment. (**D**) Example traces of SC PSC before (pre) and 60 min. after (post) electrical stimulation of Schaffer collaterals (900x @1 Hz, e-LTD). Scake bars, 100 pA, 20 ms (**E**) e-LTD caused a significant reduction of PSC peak amplitude (*n* = 7) after 60 minutes of recording compared to controls (*n* = 7). Unpaired t-test **p = 0.0012. (**F**) Examples of confocal live-imaging of an AIS during e-LTD with stable AIS length. Scale bar, 10 µm. (**G**) e-LTD does not change AIS length in control after 30 or 60 minutes of recording. Mixed-effects analysis with Dunnett's multiple comparisons test, p = 0.24 control, (*n* = 8) p = 0.44 e-LTD for the

factor treatment, $p > 0.05$ for all post-hoc multiple comparisons. (**H**) Length of AnkG, $Na_V1,2$ or $Na_V1.6$ in hippocampal cultures in control condition or after mGluR activation with DHPG. For AnkG: *t*-tests, ns $p = 0.78$, for $Na_V1.2$: Mann-Whitney test, $p = 0.052$, for $Na_V1.6$: *t*-tests, $p = 0.38$, $n > 194$ neurons, from $N = 2$ independent experiments. (**I**) DC current injection generating neuronal action potential firing for ~5 minutes ($n = 9$) reveals a stable AIS length up to 60 minutes of recording and imaging. Mixed-effects analysis with Šídák's multiple comparisons test $p = 0.118$ for the factor treatment. $p > 0.05$ for all multiple comparisons. Scale bars, 10 mV, 1 ms and 5 µm.

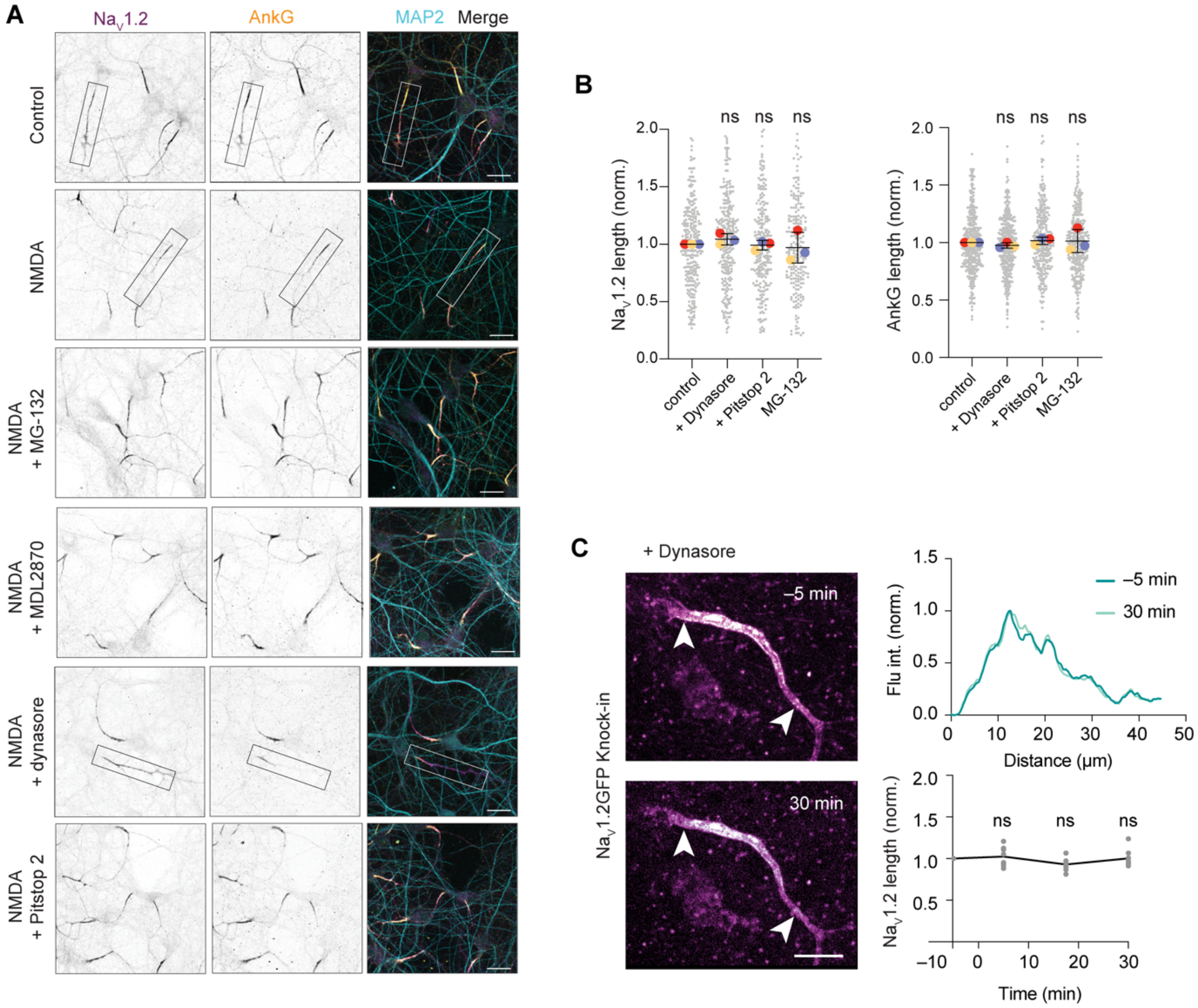

Fig. S6. Blocking clathrin-mediated endocytosis prevents NMDA-induced AIS shortening

(**A**) DIV14 hippocampal neurons in control condition, after NMDA treatment and in the presence of indicated drugs, stained for AnkG, $Na_V1.2$ and MAP2. Zooms of the boxed areas are shown in Fig. 4C. Scale bars, 30 µm. (**B**) AnkG length after treatment relative to control condition. $N = 5$ cultures, unpaired *t*-test ns, $p > 0.26$ (at least 30 neurons per condition per experiment). (**C**) Live-cell imaging of $Na_V1.2$-GFP knock-in neuron 5 min before and 30 min after treatment with Dynasore. White arrowheads point to the start and end point of the $Na_V1.2$-GFP signal. Corresponding fluorescence normalized intensities are shown on the top right, and average $Na_V1.2$-GFP length over time, and normalized to the first frame, are shown on the bottom right. $n = 8$ neurons from 2 independent experiments, Friedman test with Dunn's multiple comparison test, ns $p > 0.099$. Scale bar, 10 µm.

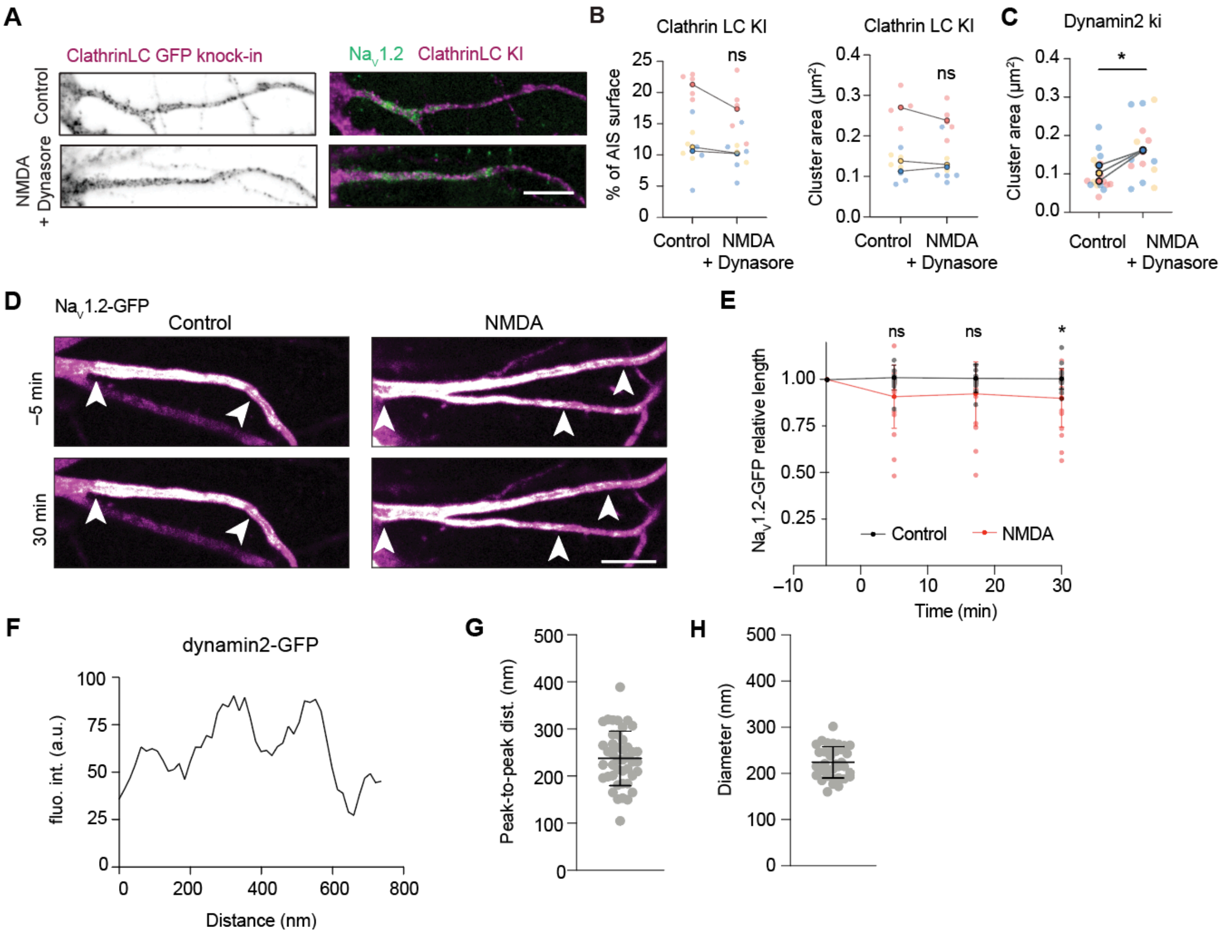

Fig. S7. Dynamin2 is recruited at the AIS during NMDA-induced plasticity

(**A**) Confocal images of the AIS of ClathrinLC-GFP knock-in neurons stained for GFP and $Na_V1.2$ in control condition and after NMDA in the presence of Dynasore. Scale bar, 10 µm. (**B**). Population analysis of clathrinLC mean surface occupancy at the AIS and cluster area. For the AIS occupancy: paired t-test, ns p = 0.24. For the cluster area: paired t-test, ns p = 0.49. *N* = 3 independent experiments, 4–5 neurons per condition per experiment. (**C**) Population analysis of the mean dynamin2 cluster area, paired t-test, *p = 0.035, *N* = 3 experiments with 4 to 7 neurons per experiment. (**D**) Live-cell imaging of $Na_V1.2$-GFP over-expressing neuron 5 min before and 30 min after treatment with NMDA or in control condition. Scale bar is 10 µm, white arrowheads point to the start and end point of the $Na_V1.2$-GFP signal. (**E**) Average $Na_V1.2$-GFP length of neurons over time, and normalized to the first frame, are shown in control condition (grey) and after NMDA application (red). *n* = 20 (control) and 19 (NMDA) neurons from 2 independent experiments. Repeated-measure two-way ANOVA. –5 min: ns, p = 0.79, 5 min: ns, p = 0.09, 17.5 min: ns, p = 0.23, 30 min: *p = 0.040. (**F**) Florescence intensity of dynamin2-GFP in a control neuron along the ROI indicated by an asterisk on Figure 5 panel E. (**G**) Average peak-to-peak distance of the autocorrelation profiles of dynamin2-GFP fluorescence intensity in control neurons. *n* = 44 ROIs from 6 neurons. (**H**) Average diameter of dynamin2-GFP endocytic structures *n* = 35 endocytic structures from 6 neurons.

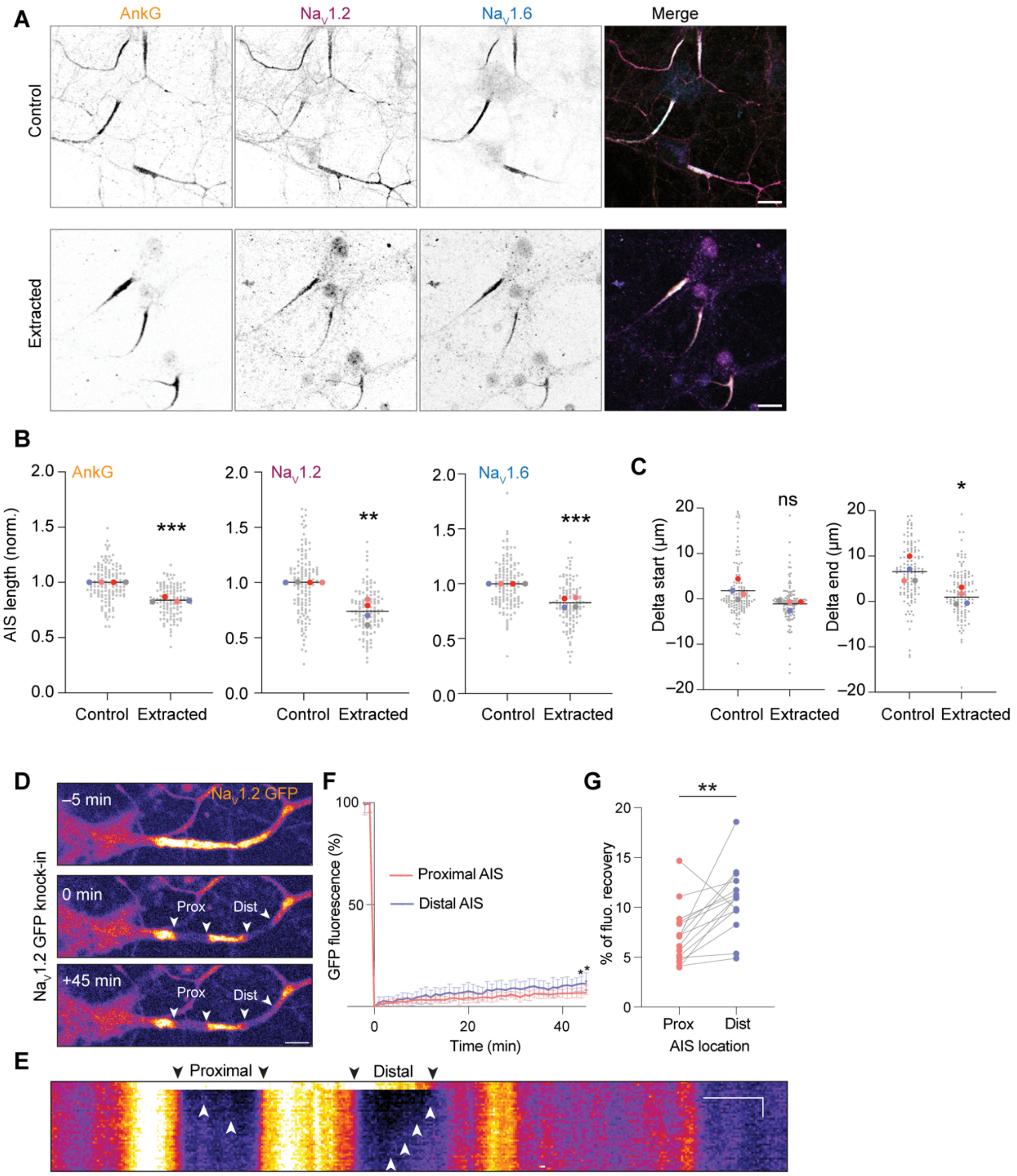

Fig. S8. Local differences in Na$_V$1.2 anchoring properties at the AIS membrane

(A) DIV14 hippocampal neurons in control condition or after Triton extraction stained for AnkG, Na$_V$1.2 and Na$_V$1.6. Scale bars are 20 µm. **(B)** Length of AnkG, Na$_V$1.2 and Na$_V$1.6 after extraction relative to the control condition. $N = 4$ experiments, Unpaired t-test, for AnkG ***p < 0.0001, for Na$_V$1.2 **p = 0.002 and for Na$_V$1.6 ***p=0.0004, at least 65 neurons per condition per experiment. (**C**) Delta start and delta end positions (Na$_V$1.2 minus Na$_V$1.6 signal onset) along the AIS in control condition and after extraction. $N = 4$ experiments, Paired t-test, for delta start ns p = 0.08, for delta end *p = 0.012 (> 60 neurons per condition per experiment). **(D)** FRAP of Na$_V$1.2-GFP knock-in neurons. Still images of a Nav1.2-GFP knock-in neuron before (–5 min), 0 min and 45 min after FRAP in the proximal and distal area, FRAP ROIs are indicated by white arrowheads. Scale bar,

5 µm. **(E)** Corresponding kymograph, white arrowheads indicate the fluorescence recovery. Scale bars, 5 µm (horizontal) and 10 min (vertical). **(F)** Average fluorescence recovery in the proximal and distal AIS of 15 neurons, from 2 independent experiments. 2-way ANOVA with Šídák's multiple comparisons test, 44 min: *p = 0.02, 45 min: *p = 0.04. **(G)** Percentage of fluorescence recovery in individual neurons in the proximal versus distal AIS, paired t-test **p = 0.002, *n* = 15 neurons from 2 independent experiments.

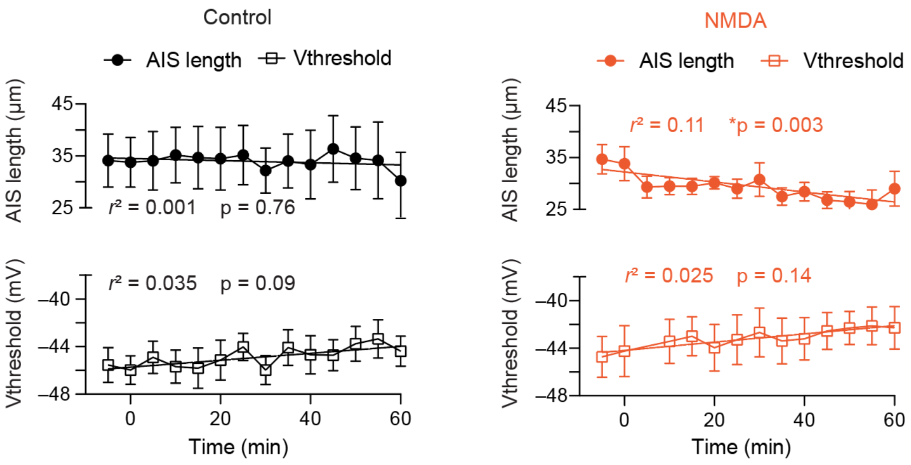

Fig. S9 Action potential voltage threshold and AIS length change over time

Top, AIS length and, bottom, voltage threshold was measured every five minutes. The R squared (r^2) and significance values of the simple linear regression fits are indicated in the graphs.

Movie S1.

Time lapse movie of a whole-cell recorded control CA1 pyramidal neurons with AnkG-GFP labeled AIS imaged every five min. from 0 (application of 5 min. APV) to 60 min. The patch pipette is visible in the top left. Imaging performed with a confocal spinning disk. Image stacks were contrast adjusted and registered over time. Scale bar 15 µm.

Movie S2.

Time lapse movie of a whole-cell recorded CA1 neuron with AnkG-GFP labeled AIS imaged every five min. from 0 (application of 3 min. NMDA followed by 5 min. APV) to 60 min. The patch pipette is visible in the top left. Imaging performed with a confocal spinning disk. Image stacks were contrast adjusted and registered over time. Scale bar 15 µm.
